# Selection of cross-reactive T cells by commensal and food-derived yeasts drives cytotoxic T_H_1 cell responses in Crohn’s disease

**DOI:** 10.1038/s41591-023-02556-5

**Published:** 2023-09-25

**Authors:** Gabriela Rios Martini, Ekaterina Tikhonova, Elisa Rosati, Meghan Bialt DeCelie, Laura Katharina Sievers, Florian Tran, Matthias Lessing, Arne Bergfeld, Sophia Hinz, Susanna Nikolaus, Julia Kümpers, Anna Matysiak, Philipp Hofmann, Carina Saggau, Stephan Schneiders, Ann-Kristin Kamps, Gunnar Jacobs, Wolfgang Lieb, Jochen Maul, Britta Siegmund, Barbara Seegers, Holger Hinrichsen, Hans-Heinrich Oberg, Daniela Wesch, Stefan Bereswill, Markus M. Heimesaat, Jan Rupp, Olaf Kniemeyer, Axel A. Brakhage, Sascha Brunke, Bernhard Hube, Konrad Aden, Andre Franke, Iliyan D. iliev, Alexander Scheffold, Stefan Schreiber, Petra Bacher

**Affiliations:** 1 Institute of Immunology, Christian-Albrechts-University of Kiel and University Medical Center Schleswig-Holstein, Kiel, Germany; 2 Institute of Clinical Molecular Biology, Christian-Albrechts-University of Kiel and University Medical Center Schleswig-Holstein, Kiel, Germany; 3 The Jill Roberts Institute for Research in Inflammatory Bowel Disease, Weill Cornell Medicine, Cornell University, New York, NY, USA; 4 Joan and Sanford I. Weill Department of Medicine, Weill Cornell Medicine, Cornell University, New York, NY, USA; 5 Department of Microbiology and Immunology, Weill Cornell Medicine, Cornell University, New York, NY, USA; 6 Department of Internal Medicine I, University Medical Center Schleswig-Holstein, Kiel, Germany; 7 Institute of Epidemiology, Christian-Albrechts-University of Kiel and popgen Biobank, University Medical Center Schleswig-Holstein, Kiel, Germany; 8 Gastroenterologie am Bayerischen Platz, Berlin, Germany; 9 Department of Gastroenterology, Rheumatology and Infectious Diseases, Charité-Universitätsmedizin Berlin, Humboldt-Universität zu Berlin and Berlin Institute of Health, Berlin, Germany; 10 Gastroenterology–Hepatology Center Kiel, Kiel, Germany; 11 Institute of Microbiology, Infectious Diseases and Immunology, Charité-Universitätsmedizin Berlin, Humboldt-Universität zu Berlin and Berlin Institute of Health, Berlin, Germany; 12 Department of Infectious Diseases and Microbiology, University of Lübeck, Lübeck, Germany; 13 Department of Molecular and Applied Microbiology, Leibniz Institute for Natural Product Research and Infection Biology—Hans Knoell Institute, Jena, Germany; 14 Friedrich Schiller Universität, Jena, Germany; 15 Institute of Microbiology, Department of Microbial Pathogenicity Mechanisms, Leibniz Institute for Natural Product Research and Infection Biology—Hans Knoell Institute, Jena, Germany

## Abstract

Aberrant CD4^+^ T cell reactivity against intestinal microorganisms is considered to drive mucosal inflammation in inflammatory bowel diseases. The disease-relevant microbial species and the corresponding microorganism-specific, pathogenic T cell phenotypes remain largely unknown. In the present study, we identified common gut commensal and food-derived yeasts, as direct activators of altered CD4^+^ T cell reactions in patients with Crohn’s disease (CD). Yeast-responsive CD4^+^ T cells in CD display a cytotoxic T helper cell (T_H_1 cell) phenotype and show selective expansion of T cell clones that are highly cross-reactive to several commensal, as well as food-derived, fungal species. This indicates cross-reactive T cell selection by repeated encounter with conserved fungal antigens in the context of chronic intestinal disease. Our results highlighted a role of yeasts as drivers of aberrant CD4^+^ T cell reactivity in patients with CD and suggest that both gut-resident fungal commensals and daily dietary intake of yeasts might contribute to chronic activation of inflammatory CD4^+^ T cell responses in patients with CD.

Inflammatory bowel diseases (IBDs) are chronic inflammatory disorders of the gastrointestinal tract, the main forms of which are CD and ulcerative colitis (UC). Control of the immune process through conventional or targeted therapies is poor in these chronic diseases, which results in lifelong morbidity, strongly reduced quality of life and high medical costs. Aberrant immune reactions, particularly CD4^+^ T cell responses, against members of the intestinal microbiota are considered a causal or driving factor for an IBD. However, the high diversity of the microbiome has largely prevented identification of disease-driving, microbial T cell targets and the corresponding microorganism-specific, pathogenic T cell phenotypes in IBDs. Furthermore, owing to the complexity of IBDs, pathogenic CD4^+^ T cell phenotypes and disease-relevant antigens may also vary for different patient subgroups.

T_H_17 cells are regarded as central orchestrators of homeostatic immune responses against microorganisms. Dysregulated T_H_17 cell responses, in turn, are thought to contribute to intestinal inflammation and increased levels of interleukin (IL)-17A-producing cells can be found in the intestinal mucosa of patients with IBD^1,2^. However, neutralization of IL-17 has been clinically ineffective in CD and even caused exacerbation in some patients^3,4^, suggesting that IL-17A could play a protective rather than a pathological role in CD or in a subgroup of these patients. In this regard, T_H_17 cell plasticity and the development of pathogenic T_H_17 cell subsets that coexpress inflammatory cytokines, such as interferon (IFN)-γ or granulocyte-macrophage colony-stimulating factor (GM-CSF), might be important determinants for a protective versus a pathogenic role for T_H_17 cells^5^. In addition, T_H_1 cells expressing the signature cytokine IFN-γ are consistently found to be increased in the intestine of patients with CD, but not those with UC^6^. Thus, deciphering the disease-relevant microbial antigens and unique features of pathogenic, microorganismspecific, CD4^+^ T cell responses could give rise to the development of targeted therapies.

Most studies to date have addressed alterations in the intestinal bacterial microbiota in IBDs and few studies have analyzed bacteria-specific CD4^+^ T cell responses^7–14^. Although bacteria are more abundant organisms in the gastrointestinal tract, fungi are an integral, but largely neglected, part of the human intestinal microbiota. Only recently has the important role of individual fungal species on human health and immunopathology come into focus^15–22^. In particular, the intestinal commensal fungus *Candida albicans* has been identified as a major modulator of the human immune system by inducing homeostatic T_H_17 cell responses^15,23^, as well as intestinal immunoglobulin (Ig)A^1,22^ and systemic IgG antibodies^16^. It is still unclear whether *C. albicans-specific* T_H_17 cells contribute to intestinal inflammation in patients with IBD or are recruited to the site of inflammation to foster healing. Most interestingly, a high prevalence of *anti-Saccharomyces cerevisiae* antibodies (ASCAs) has been consistently reported in the serum of patients with CD. Although *S. cerevisiae* mannans were usually used as a binding antigen in these assays, further studies have demonstrated that ASCAs also bind to mannans from other fungi, including *C. albicans*^24^. In addition to *C. albicans*, the human intestine also harbors a plethora of other commensal, as well as food-derived, fungi^25–27^. Whether these fungal species can also actively modulate human immune responses has not yet been addressed.

In the present study, we show that CD4^+^ T cell responses against commensal and food-derived yeasts are strongly increased in blood and inflamed tissue of patients with CD, but not those with UC. These yeast-reactive CD4^+^ T cells display cytotoxic T_H_1 cell effector functions, whereas *C. albicans-specific* T_H_17 cell responses remain largely unchanged. Cytotoxic T_H_1 cells are clonally expanded and highly cross-reactive to several commensal and food-derived yeast species, indicating their selection by chronic encounter with conserved antigens that are present in different yeasts. Our data identify commensal and dietary yeasts as drivers of aberrant CD4^+^ T cell reactivity in patients with CD and suggest that repeated encounter with conserved antigens present in different microbial species may lead to the expansion and chronic activation of cross-reactive, yeast-responsive, CD4^+^ T cells. This could reveal a general mechanism of how selection of cross-reactive T cells may allow adaptive immunity to cope with the enormous diversity of microbial antigens which, if not properly regulated, could come at the expense of fostering chronicity and contribution to therapy resistance in IBD due to persistent antigen activation.

## Results

### CD4^+^ T cell responses against yeasts are increased in CD

**Extended Data Fig. 1:**
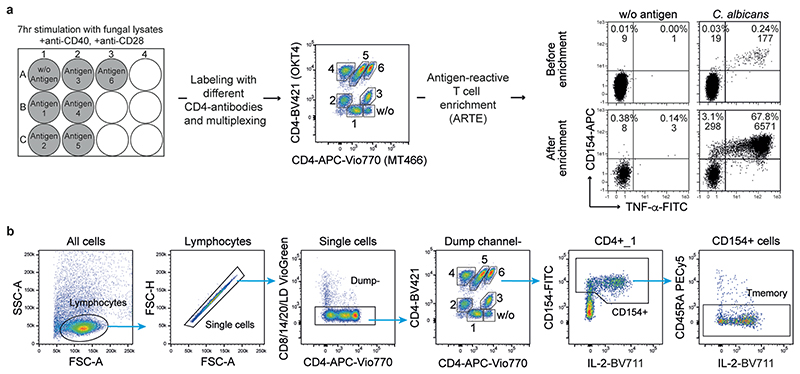
Antigen-reactive T cell enrichment (ARTE). (**a**) Experimental set-up of ARTE in combination with multiplexing of differently stimulated samples. PBMCs were stimulated with different microbial lysates for 7 hours. Cells were labeled with a CD4-antibody-based fluorescent barcode using different anti-CD4 clones and mixed. Reactive CD154+ T cells were enriched by ARTE. Dot plot examples show *C. albicans* lysate-stimulated cells before enrichment and after magnetic CD154+ enrichment from 2x10^7^ PBMCs. Percentage of CD154+ cells within CD4+ T cells and absolute cell counts are indicated. (**b**) Gating strategy following enrichment of CD154+ cells via ARTE.

**Extended Data Fig. 2:**
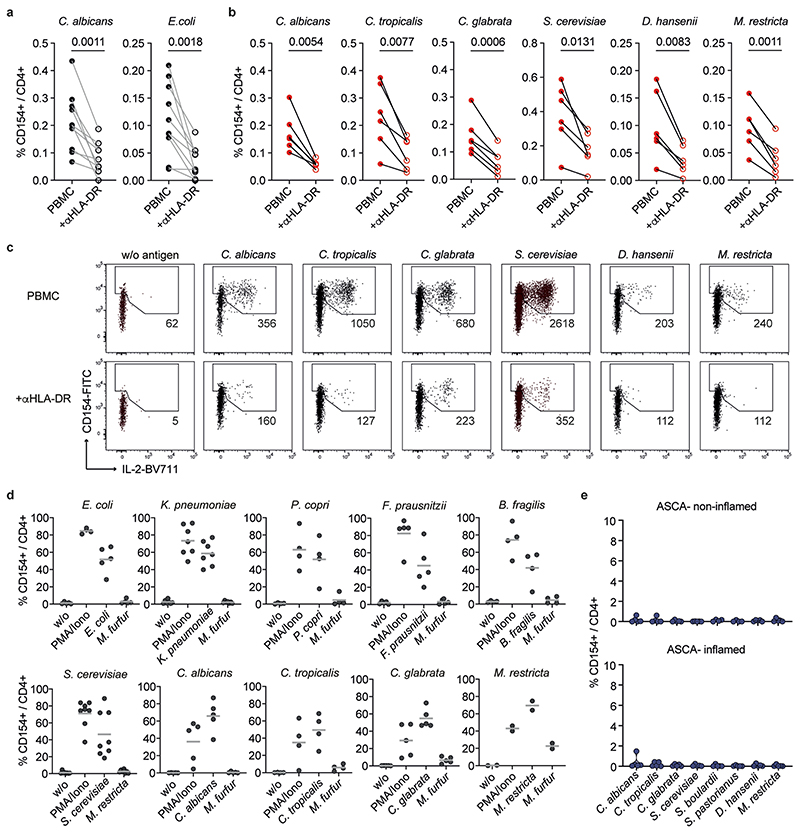
Specificity of microbial lysate induced CD154 expression. (**a**, **b**) Frequencies of microbial lysate stimulated CD154+ T cells stimulated in presence or absence of an anti-HLA-DR antibody of (a) healthy donors (n = 10) and (**b**) CD patients (n = 6). (**c**) Representative flow plots showing CD154 induction in presence or absence of an anti-HLA-DR antibody. Numbers of enriched CD154+ cells are indicated. (**d**) Following stimulation with microbial lysates, CD154+ cells were FACS-purified, expanded for several weeks and restimulated with the specific or unrelated microbial lysates in the presence of autologous antigen presenting cells. PMA-Ionomycin stimulation was used as positive control. Percentage of CD154+ TNFα+ cells within CD4+ is indicated (*E. coli*, *F. prausnitzii*, *C. albicans*, *C. glabrata* n = 5; *K. pneumoniae* n = 7; *P. copri*, *B.fragilis*, *C. tropicalis* n = 4; *S. cerevisiae* n = 8; *M. restricta* n = 2). (**e**) Total CD4+ memory T cells were isolated from biopsies of inflamed and uninflamed intestinal tissue of ASCA- CD patients (n = 5). Cells were seeded at 200 cells/well in multiple wells, expanded and re-stimulated with fungal lysates in presence of autologous APCs. Mean frequencies of reactive CD154+ cells for all wells per patient are shown. Each symbol in (**a**, **b**, **d**, **e**) represents one individual donor. Truncated violin plots with quartiles and range are shown in (e). Statistical differences: two-tailed paired t test in (**a**, **b**).

To identify microbial species driving pathogenic T cell responses in IBDs, we analyzed the CD4^+^ T cell reaction against common bacterial and fungal species of the human intestinal microbiome in patients with IBD and healthy donors (see Extended Data Tables 1 and 2 for demographic data). Peripheral blood mononuclear cells (PBMCs) were stimulated ex vivo for 7 h with whole bacterial and fungal lysates. The differentially stimulated cells were labeled with CD4 antibody-based fluorescent barcodes, allowing multiplexed analysis of T cell reactivity against different species (Extended Data Fig. 1a). Microorganism-reactive CD4^+^ T cells were detected based on magnetic enrichment of CD154 (CD40L)-expressing cells (antigen-reactive T cell enrichment (ARTE))^15,28^ (Extended Data Fig. 1a,b). Specificity of CD154^+^ induction by microbial lysates was confirmed by blocking antigen presentation with a human leukocyte antigen (HLA)-DR antibody (Extended Data Fig. 2a–c) and by restimulation of expanded CD154^+^ T cells with specific or unrelated antigen lysates (Extended Data Fig. 2d).

**Fig. 1:**
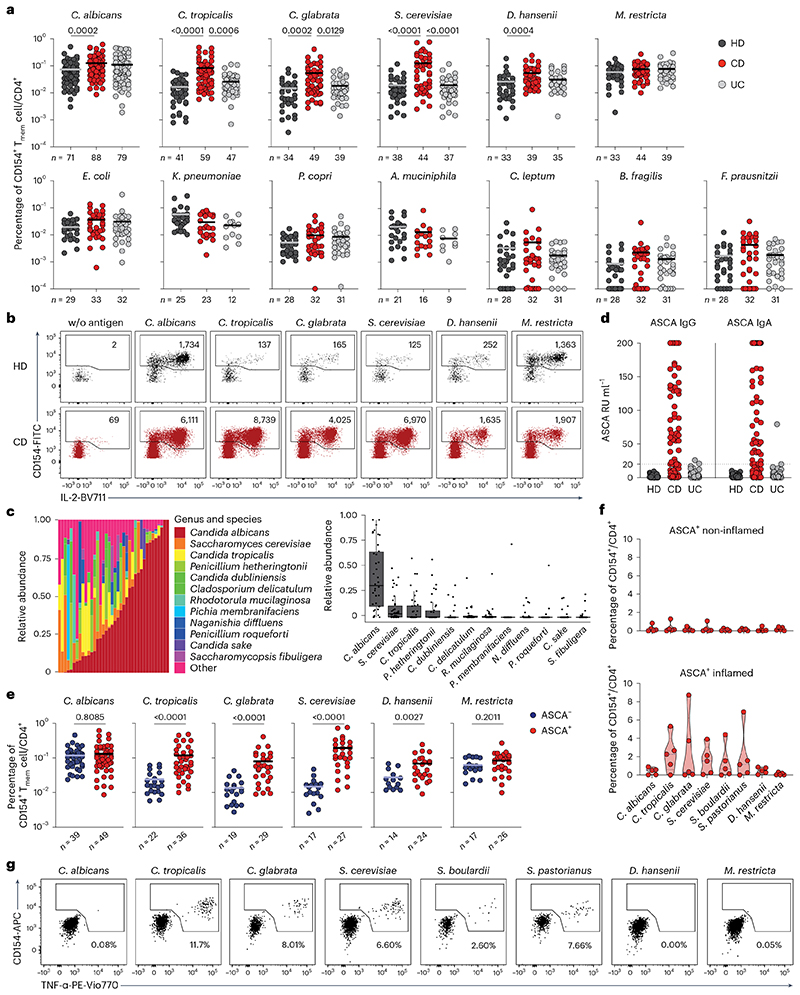
Altered CD4^+^T cell reactivity against fungal microorganisms in CD patients. **a**, Frequencies of reactive CD154^+^CD45RA^−^CD4^+^ T_mem_ cells against whole microbial lysates in healthy donors and patients with an IBD: healthy donors (HD, black dots), CD (red dots) and UC (gray dots). The letter *n* indicates the number of individual donors specified below each group. The *P* values are given at the top of each figure. **b**, Dot plot examples for the ex vivo detection of microorganism-reactive CD4^+^ T cells by ARTE. Absolute cell counts after magnetic CD154^+^enrichment from 1× 10^7^ PBMCs are indicated. w/o, without. **c**, ITS-seq and analysis of stool samples from patients with CD (*n* = 38). The relative abundance of the top 12 fungal species identified by this analysis is shown. Less abundant or less represented fungal species are grouped under ‘other’, **d**, Serum anti-IgG and IgA ASCA concentrations in healthy donors and patients with IBD (HD, *n* = 46; CD, *n* = 88; UC, *n* = 73). Sera were considered positive if their activity was >20 relative units (RU) per ml. Both single-positive IgA or IgG samples and double-positive samples classified donors who are ASCA^+^. **e**, Frequencies of fungus-reactive CD4^+^ T cells in patients with CD, according to their ASCA status. **f**, Total CD4^+^ T_mem_ cells isolated from biopsies of inflamed and uninflamed intestinal tissue of patients with CD who are ASCA^+^ (*n* = 5). Cells were seeded at 200 cells per well in multiple wells, and expanded and restimulated with fungal lysates in the presence of autologous APCs. Mean frequencies of reactive CD154^+^cells for all wells per patient are shown. **g**, Dot plot examples for restimulation of expanded CD4^+^ T_mem_ cells from an inflamed biopsy. The percentage of CD154^+^ TNF^+^cells within CD4^+^ is indicated. Each symbol in **a**, **d**, **e** and **f** represents one individual donor and horizontal lines in **a** and **e** indicate the mean. Box-and-whisker plots are shown in **c** with center lines representing the median, box limits 25% (lower) and 75% (upper) quartiles and whiskers the minimum/maximum. Truncated violin plots with quartiles and range are shown in **f**. Statistical differences were obtained using the Kruskal–Wallis test with Dunn’s post hoc test in **a** with only significant differences shown; the two-tailed Mann–Whitney *U*-test was used in **e**.

Circulating CD45RA^−^ memory T cells (T_mem_ cells) reactive against different microbial species were detected with variable frequencies (range 0.0001–0.76%) (Fig. 1a,b). Although *C. albicans*, the most abundant intestinal fungal commensal^20,29^ (Fig. 1c), elicited the strongest reactivity in healthy individuals, we observed only a slight increase in reactive T_mem_ cells in patients with CD. In sharp contrast, reactivity against other less abundant *Candida* spp., as well as against the food-associated yeast *Saccharomyces cerevisiae* (Fig. 1c), was dramatically elevated in patients with CD, but not those with UC (Fig. 1a,b). No significant differences were observed against the phylogenetically distant yeast *Malassezia restricta* or the analyzed bacterial species (Fig. 1a,b). Together this indicates an altered antifungal immune response to gut mycobiota in patients with CD. However, we also noticed a high heterogeneity in the frequencies of antifungal T cells between different patients with CD (Fig. 1a). ASCAs can be detected in the serum of 50–60% of patients with CD and are used as a biomarker of disease severity^30^. When stratifying our cohort of patients with CD according to their ASCA immunoglobulin IgG/IgA status (Fig. 1d), strikingly, increased yeast-reactive T cell responses were detected in ASCA^+^, but not ASCA^−^, patients with CD (Fig. 1e). In contrast, the strong T_mem_ cell response to *C. albicans* was independent of the ASCA status.

These results indicate that, in addition to augmented T_mem_ cell responses to commensal *C. albicans* in patients with CD^15^, a cohort of ASCA^+^ patients develops unexpected T cell reactivity to less represented fungal commensals and food-derived yeast species that so far have not been described as major inducers of T cell reactivity in humans.

### Yeast-reactive CD4^+^ T cells are enriched in inflamed mucosa

**Extended Data Fig. 3:**
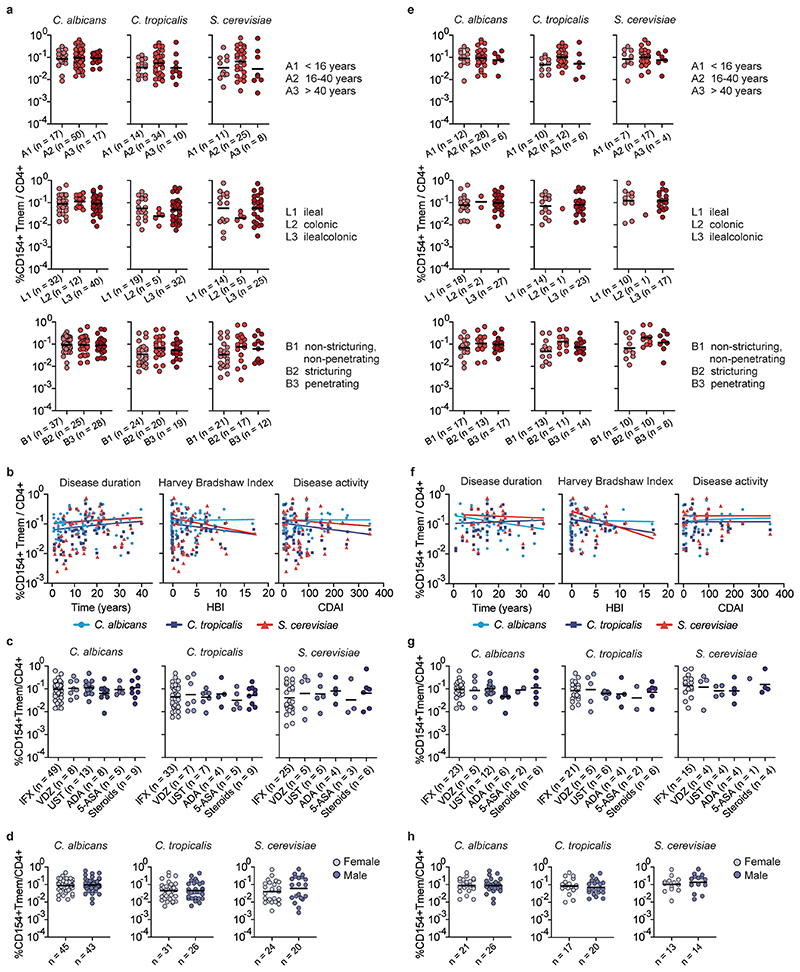
Correlation of T cell data with clinical parameters. Reactive CD4+ T cell frequencies against *C. albicans*, *C. tropicalis* and *S. cerevisiae* were correlated to different clinical parameters in all CD patients (**a**-**d**) or ASCA+ CD patients (**e**-**h**). (**a**, **e**) Influence of age at diagnosis, disease localization or behavior, based on Montreal classification, on fungus-reactive T cell frequencies. n indicates the number of individual donors specified below each group. (**b**, **f**) Spearman correlation of yeast-reactive CD4+ T cell frequencies with disease duration or activity, as determined by Harvey-Bradshaw Index (HBI) or Crohn’s disease activity index (CDAI). (**b**, *C. albicans* n = 88, *C. tropicalis* n = 59, *S. cerevisiae* n = 44; **f**, *C. albicans* n = 50, *C. tropicalis* n = 38, *S. cerevisiae* n = 28). (c, g) Yeast-reactive T cell frequencies according to different treatments. IFX, Infliximab; VDZ, Vedolizumab; UST, Ustekinumab; ADA, Adalimumab; 5-ASA, 5-aminosalicylic acid. n indicates the number of individual donors specified below each group. (**d**, **h**) Yeast-reactive T cell frequencies according to sex. n indicates the number of individual donors specified below each group. Each symbol in **a**-**h** represents one individual donor. Horizontal lines indicate geometric mean values in (**a, c, d, e, g, h**).

We next analyzed whether yeast-responsive T cells are also present at the site of intestinal inflammation. As the cell numbers obtained from intestinal biopsies are too low for direct analysis by ARTE, we expanded total CD4^+^ T cells from inflamed and noninflamed gut biopsies polyclonally and restimulated them with various fungal lysates. Yeast-reactive T cells were selectively present in inflamed gut tissue from patients who were ASCA^+^, but not in noninflamed tissue from the same individuals (Fig. 1f,g), or in tissue from patients with CD who are ASCA^−^ (Extended Data Fig. 2e). Notably, this applied also to other *Saccharomyces* spp., such as *Saccharomyces pastorianus* or the probiotic yeast *Saccharomyces boulardii*, which is sometimes used as a treatment supplement for IBD^31^, whereas reactivity to *C. albicans* was low (Fig. 1f). We further examined whether the increased T cell reactivity against yeasts might be influenced by disease state or duration. There were no significant correlations of yeast-reactive T cell frequencies with disease duration, medication or activity, as determined by the Harvey–Bradshaw index and the CD activity index (Extended Data Fig. 3).

In summary, these data reveal an unexpected increase of CD4^+^ T cell reactivity against intestinal commensal and food-derived yeasts in blood and inflamed tissue in patients with CD who are ASCA^+^. Although *C. albicans* dominates the antifungal T cell response in healthy individuals and *C. albicans-reactive* T cells are still highly present in patients with CD, the increased T cell reactivity in patients with CD who are ASCA^+^ was mainly directed against *non-albicans Candida* spp., as well as food-associated *Saccharomyces* spp.

### Yeast-reactive CD4^+^ T cells show increased IFN-γ production

**Extended Data Fig. 4:**
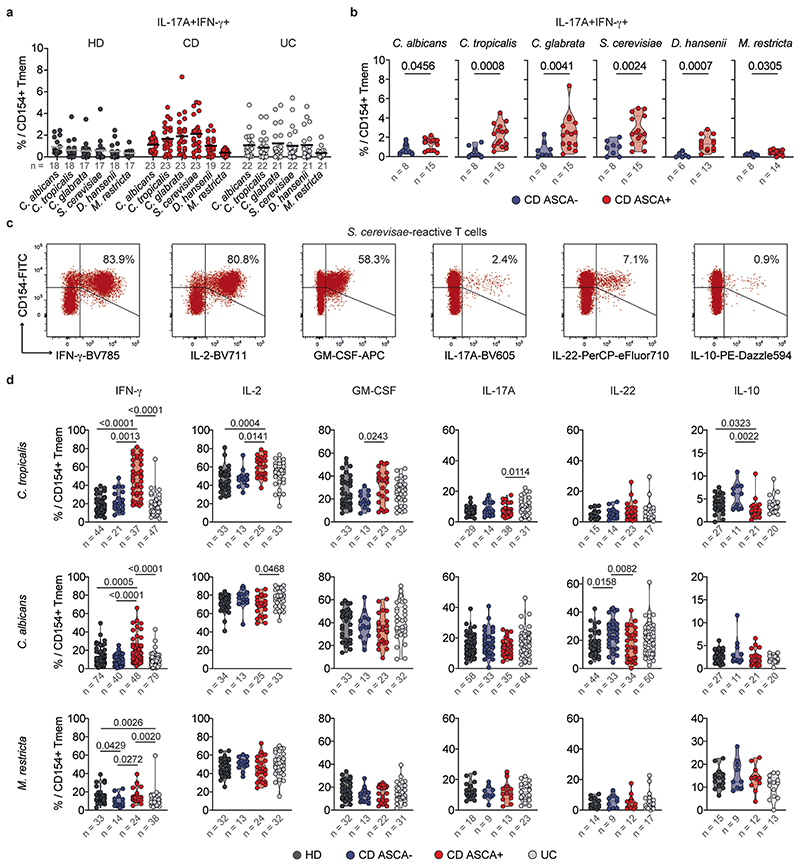
Cytokine production of yeast-reactive CD4+ T cells. (**a**) Proportion of IL-17A+IFN-γ+ cells reactive against the different fungal antigens. n indicates the number of individual donors specified below each group. (**b**) Proportion of IL-17A+IFN-γ+ cells in CD patients according to their ASCA status. n indicates the number of individual donors specified below each group. (**c**) Example plots for ex vivo cytokine production of *S. cerevisiae*-reactive Tmem following ARTE. Percentages of cytokine positive cells within CD154+ Tmem are indicated. (**d**) Cytokine production of *C. tropicalis*, *C. albicans* and *M. restricta*-reactive T cells in healthy donors and IBD patients. n indicates the number of individual donors specified below each group. Each symbol in (**a**, **b**, **d**) represents one individual donor, truncated violin plots with quartiles and range are shown in (**b**, **d**). Statistical differences: two-tailed Mann-Whitney test in (**b**); Kruskal-Wallis test with Dunn’s post hoc test in (**d**), only significant differences are shown.

**Fig. 2:**
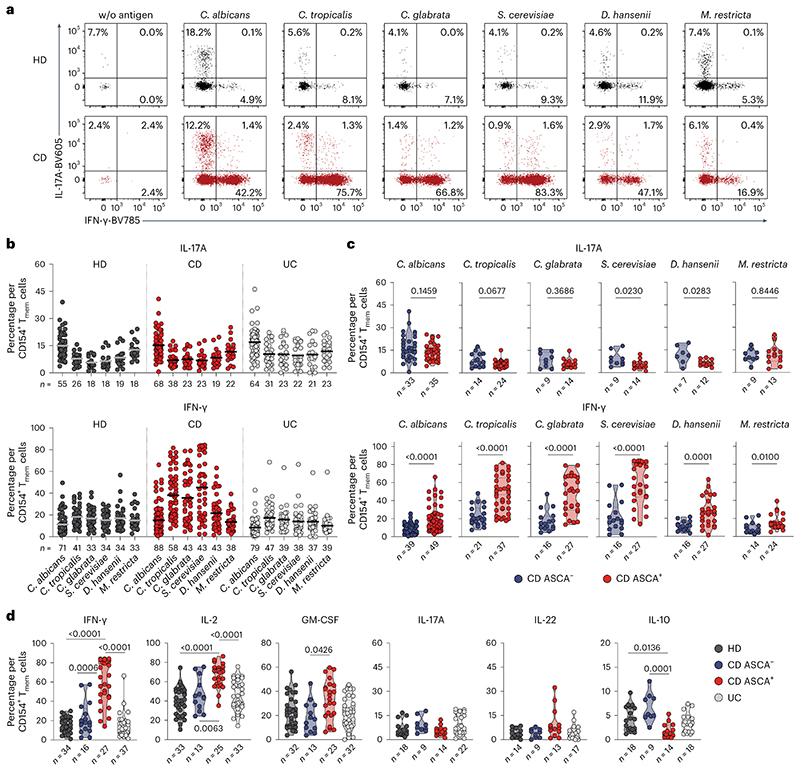
Enhanced T_H_1 cell but not T_H_17 cell responses against yeasts in patients with CD. **a**,**b**, Ex vivo cytokine production of fungus-reactive T_mem_ cells following ARTE. **a**, IL-17A and IFN-γ staining of yeast-reactive CD154^+^ T cells. **b**, Statistical summary in healthy donors and patients with IBD. **c**, IL-17A and IFN-γ production of yeast-reactive CD4^+^ T cells in patients with CD according to their ASCA status. **d**, Cytokine production of*S. cerewsiae-*reactive T cells in healthy donors and patients with IBD. Each symbol in **b**, **c** and **d** represents one individual donor and horizontal lines indicate the mean. Truncated violin plots with quartiles and range are shown in **c** and **d**. Statistical differences (shown in figures) were obtained using the two-tailed Mann–Whitney *U*-test in **c** and the Kruskal–Wallis test with Dunn’s post hoc test in **d**; only significant differences are shown.

Intestinal inflammation in IBD has been linked to aberrant T_H_17 cell responses and the development of pathogenic T_H_17 cells which coproduce inflammatory cytokines such as IFN-γ^1,2^. We recently showed that, within a number of species found in the human mycobiome, *C. albicans* is the major driver of fungus-reactive T_H_17 cells in humans^15^. However, we detected no significant differences of fungus-reactive IL-17A production in patients with IBD compared with healthy donors (Fig. 2a,b). In contrast, CD4^+^ T cells reactive against the different yeast species, including *C. albicans*, showed a massively increased IFN-γ production in patients with CD (Fig. 2a,b), which was again strongly enhanced in individuals who are ASCA^+^ compared with those who are ASCA^−^ (Fig. 2c).Just as for the frequencies (Fig. 1), the increased IFN-γ production was much more pronounced for *non-albicans Candida* spp. and especially for *S. cerevisiae*. We observed only low coexpression of IFN-γ and IL-17A (Fig. 2a), indicating selective T_H_1 cell expansion rather than T_H_1 cell-like modulation of pre-existing T_H_17 cells. However, small populations (≤5%) of IL-17A^+^IFN-γ^+^ coproducing cells were also increased against *Candida* spp. and *S. cerevisiae* (Extended Data Fig. 4a,b). Further characterization of yeast-reactive T cells revealed enhanced production of several inflammatory cytokines in response to *S. cerevisiae* and *Candida tropicalis*, including IL-2 and GM-CSF, whereas IL-10 production was significantly reduced in patients with CD who are ASCA^+^ (Fig. 2d and Extended Data Fig. 4c,d). Again, we observed no differences of *M. restricta*-reactive T cells (Extended Data Fig. 4d).

Collectively, these data show strongly increased inflammatory T_H_1 cell responses against *Candida* and *Saccharomyces* yeasts in patients with CD who are ASCA^+^ and only a minor development of IL-17A^+^IFN-γ^+^ cells. In contrast, the proportion of IL-17A producers within *C. albicans-specific* T cells was conserved and remained largely unaffected by the disease.

### Yeast-reactive T_H_1 cells have a cytotoxic signature

**Extended Data Fig. 5:**
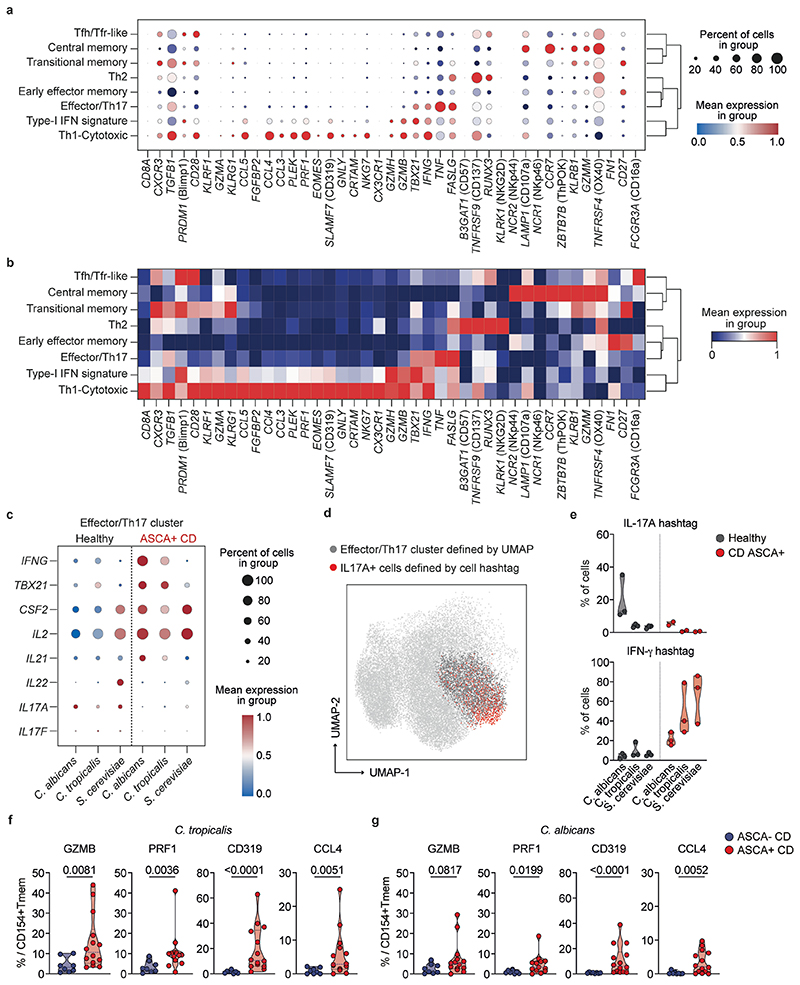
Yeast-responsive Th1 cells express several cytotoxicity-associated markers. (**a**) Dot plot visualization showing the expression of cytotoxicity-associated^32^ genes in each T cell cluster. Colors represent the normalized mean expression, and size indicates the proportion of cells expressing the respective genes. (**b**) Heatmap showing the expression of selected genes in each T cell cluster. Colors represent the Z-score-normalized expression amounts. (**c**) Dot plot visualization showing the expression of selected marker genes within the effector/Th17 cluster for healthy donors and CD patients according to antigen specificity. Colors represent the normalized mean expression levels, and size indicates the proportion of cells expressing the respective genes. (**d**) IL-17A producing cells were labeled with hashtag antibodies prior to scRNA sequencing (see Methods) and are plotted as overlay with the effector/Th17 cluster. These data show that only a subset of cells within this cluster has a Th17 signature. (**e**) Proportion of IFN-γ+ or IL-17A+ cells labeled with hashtag antibodies for individual donors and specificities (healthy n = 3, CD n = 3). (f, g) Expression of cytotoxic markers in (**f**) *C. tropicalis* and (**g**) *C. albicans*-reactive T cells of CD patients according to their ASCA status. (ASCA- n = 8; ASCA+ n = 14). Each symbol in (**f**, **g**) represents one individual donor. Truncated violin plots with quartiles and range are shown in (**f**, **g**). Statistical differences: Two-tailed Mann-Whitney test in (**f**, **g**).

**Fig. 3:**
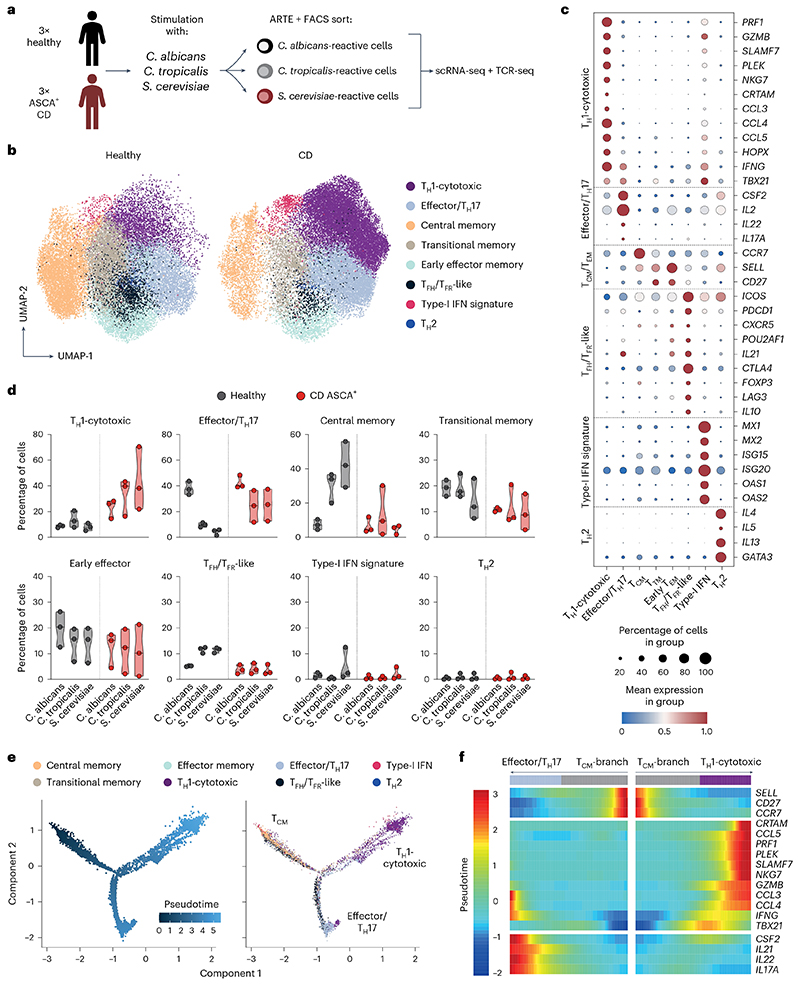
Yeast-reactive CD4^+^ cells from patients with CD have a cytotoxic phenotype. **a**, Overview of the experimental design. PBMCs of each of three healthy donors and patients with CD who were ASCA^+^ were stimulated with *C. albicans, C. tropicalis* and *S. cerevisiae* lysates. Reactive CD154^+^ T_mem_ cells were FACS purified (see Methods for sorting) and analyzed by scRNA- and TCR-seq. **b**, UMAP visualization showing the subset composition of yeast-reactive CD4^+^ T cells colored by different gene expression clusters. **c**, Dot plot visualization showing the expression of selected marker genes in each T cell cluster. Colors represent the normalized mean expression levels and size indicates the proportion of cells expressing the respective genes (T_cm_ cell, central memory cell; T_TM_ cell, transitional memory cell; T_EM_ cell, effector memory cell; T_FH_, cell follicular helper cell; T_FR_ cell, follicular regulatory cell). **d**, Proportion of cells within each T cell cluster for individual donors (healthy, *n* = 3; CD, *n* = 3). **e**, Trajectory plot of cells ordered along pseudotime (left) and overlaid with their cluster identity (right). **f**, Heatmap depicting gene expression of cluster-defining genes along the trajectory branches identified in **e**. Each symbol in **d** represents one individual donor, and truncated violin plots with quartiles and range are shown.

To obtain deeper insights into the cellular composition of yeast-reactive T cells and their functional capacities, we performed single-cell RNA sequencing (scRNA-seq) of ex vivo FACS-purified, *C. albicans-, C. tropicalis-* and *S. cerevisiae*-reactive CD154^+^ T_mem_ cells from three patients with CD who are ASCA^+^ and three healthy donors (Fig. 3a). In total, we sequenced 15,012 *C. albicans*-, 21,623 *C. tropicalis*- and 23,495 *S. cerevisiae*-reactive CD4^+^ T cells. Within these cells, we identified eight clusters with a distinct transcriptional profile based on Leiden unsupervised clustering (Fig. 3b,c). Among these clusters, we detected again a dominant T_H_1 cell population (marker genes *IFNG* and *TBX21)* that was additionally characterized by expressing high levels of cytotoxic marker genes (for example, *PRF1, GZMB* and *SLAMF7)* (Fig. 3c). In fact, cells within this cluster expressed a number of genes previously described for cytotoxic CD4^+^ T cells that arise in viral infections or cancer^32^ (Extended Data Fig. 5a,b). Furthermore, these cells expressed high levels of *HOPX*, encoding a transcription factor that has been associated with chronic T cell activation^33,34^. The T_H_1 cell-cytotoxic cluster was strongly increased in patients with CD compared with healthy donors (Fig. 3b,d). We also noticed the increase of an effector cluster in *C. tropicalis-* and *S. cerevisiae-reactive* cells from patients with CD expressing low levels of T_H_17 cell signature genes *(IL17A* and *IL22*), but also of T_H_1 cell marker genes *IFNG* and *TBX21*, as well as *CSF2* and *IL2* (Fig. 3c,d). Discrimination of healthy and patient-derived cells in this cluster revealed that CD-derived cells did not upregulate T_H_17 cell genes, but rather several other inflammatory genes defining this cluster (that is, *IFNG*, *TBX21*, *CSF2* and *IL2*) (Extended Data Fig. 5c). This is in line with the low proportion of IL-17A protein-expressing cells (Fig. 2b) and was further confirmed by direct hashtag antibody labeling of IL-17A protein-secreting T cells, which did not increase in patients with CD (Extended Data Fig. 5d,e). The other identified gene clusters either remained unchanged or were reduced in patients with CD versus controls or in general comprised only a small proportion (≤5%) of cells (Fig. 3d).

Development of T_H_17 cells into pathogenic IL-17A^+^IFN-γ^+^ coproducing cells or even a complete conversion of T_H_17 cells into T_H_1 cells has been suggested to occur in chronic inflammation^5,34^. Based on intracellular cytokine staining, we observed only a minor proportion of cells coexpressing IFN-γ and IL-17A cytokines (Fig. 2a). To further analyze the developmental origin of the yeast-reactive cytotoxic T_H_1 cells (T_H_1-CTLs), we next performed a pseudotime analysis, which reveals pseudo-temporal ordering of cells according to gene expression similarity. Using the Monocle algorithm^35^, we identified two major trajectories, starting from central memory cells (T_CM_ cells) to either the effector/T_H_17 cell cluster or the T_H_1-CTL lineage (Fig. 3e). This supports the conclusion that the T_H_1-CTLs do not primarily convert from T_H_17 cell precursors. It is interesting that expression of *IFNG* and *TBX21* preceded the expression of cytotoxic marker genes along the T_H_1-CTL branch, pointing to a progressive acquisition of cytotoxic effector functions in T_H_1 cells over time (Fig. 3f).

Together, our data reveal that, in CD, but not under a steady state, exposure to yeast antigens results in strong expansion of yeast-reactive T_H_1 cells with a cytotoxic signature.

### T_H_1-CTLs have killing ability for IECs

**Fig. 4:**
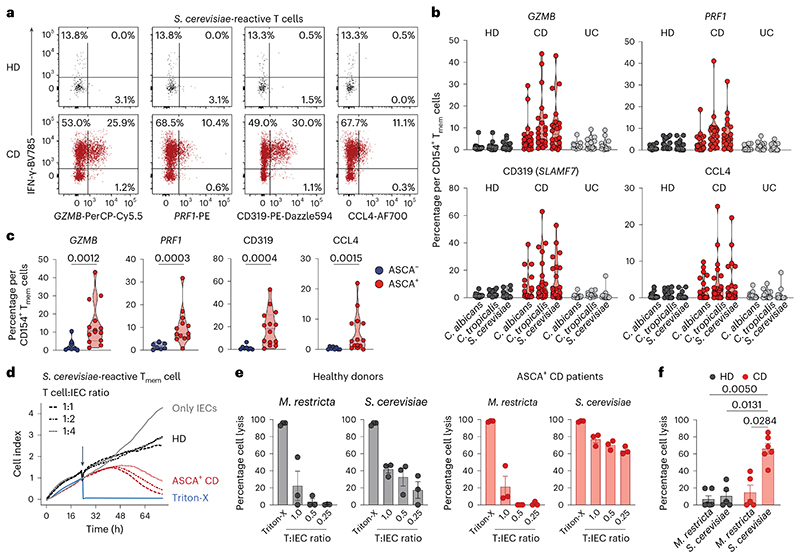
Yeast-responsive T_H_1-CTLs from patients with CD have potent killing ability for IECs. **a**, Ex vivo staining of cytotoxic markers (GZMB, PRFl, CD3l9 and CCL4) and IFN-γ of *S. cerevisiae*-reactive CD154^+^ T_mem_ cells. **b**, Statistical summary (HD, *n* = 16; CD, *n* = 22; UC, *n* = 15). **c**, Expression of cytotoxic markers in *S. cerevisiae*-reactive T cells of patients with CD according to their ASCA status (ASCA^−^, *n* = 8; ASCA^+^, *n* = 14). **d**, Real-time monitoring of cytotoxicity by electrical impedance measurement. Fungus-reactive CD154^+^ T_mem_ cells were incubated with primary IECs at the indicated T cell-to-target ratios (T:IECs) and IEC killing was monitored via changes in the cell index. Gray line: IECs without added T cells; blue line: IECs after adding the detergent Triton X-100 (positive control for maximal lysis); black lines: T cells from an HD; red lines: T cells from a patient with CD who was ASCA^+^. **e**, Summary of T cell-mediated killing after 35 h of coculture for *M. restricta-* and *S. cerevisiae*-reactive T_mem_ cells at different T cell-to-IEC ratios (healthy, *n* = 3; ASCA^+^ CD, *n* = 3). **f**, Statistical summary of T cell-mediated killing at a T cell-to-IEC ratio of 1:4 (HD *S. cerevisiae*, *n* = 5; *M. restricta*, *n* = 6; CD *S. cerevisiae*, *n* = 6, *M.restricta n* = 5). Each symbol in **b**, **c**, **e** and **f** represents one donor. Truncated violin plots with quartiles and range are shown in **b** and **c**. Error bars indicate mean and s.e.m. in **e** and **f**. Statistical differences were obtained with the two-tailed Mann–Whitney *D*-test in **c** and the Kruskal–Wallis test with Dunn’s post hoc test in **f**. Statistical differences to *S. cerevisiae*-reactive cells from patients with CD are indicated.

We confirmed the protein expression of cytotoxic markers by yeast-reactive CD4^+^ T_H_1 cells in patients with CD by flow cytometry (Fig. 4a,b). Again, T_H_1-CTLs were present in patients with CD who were ASCA^+^, but not in those who were ASCA^−^ (Fig. 4c and Extended Data Fig. 5f,g). To further investigate the killing capacity of yeast-reactive T_H_1-CTLs, we set up an in vitro cytotoxicity assay (Fig. 4d). CD154^+^ T_mem_ cells reactive to *S. cerevisiae* or *M. restricta*, used as a control antigen, were ex vivo isolated from healthy individuals and patients with CD who were ASCA^+^ and cocultured with allogeneic primary intestinal epithelial cells (IECs) in different ratios. T cells and IECs were cross-linked by the addition of *Staphylococcus* enterotoxin B (SEB) and the killing of the IECs was measured by real-time cell analysis. In line with their cytotoxic signature, *S. cerevisiae*-reactive CD4^+^ T cells from patients with CD who were ASCA^+^, but not from healthy donors, lysed IECs in a dose-dependent manner. No cytotoxicity was observed when *M. restricta-reactive* CD4^+^ T cells were used (Fig. 4e,f).

These data confirm that yeast-responsive T_H_1 cells of patients with CD who are ASCA^+^ have cytotoxic effector functions that result in potent killing ability of IECs in vitro. IECs have been reported to function as nonconventional antigen-presenting cells (APCs), presenting antigens for intestinal microorganisms^36^. It is therefore tempting to speculate that yeast-reactive T_H_1-CTLs might promote killing of IECs in vivo and thus could be involved in intestinal barrier deterioration in patients with CD, which needs further investigation.

### Yeast-reactive CD4^+^ T cells are altered in IBD-FDRs

**Extended Data Fig. 6:**
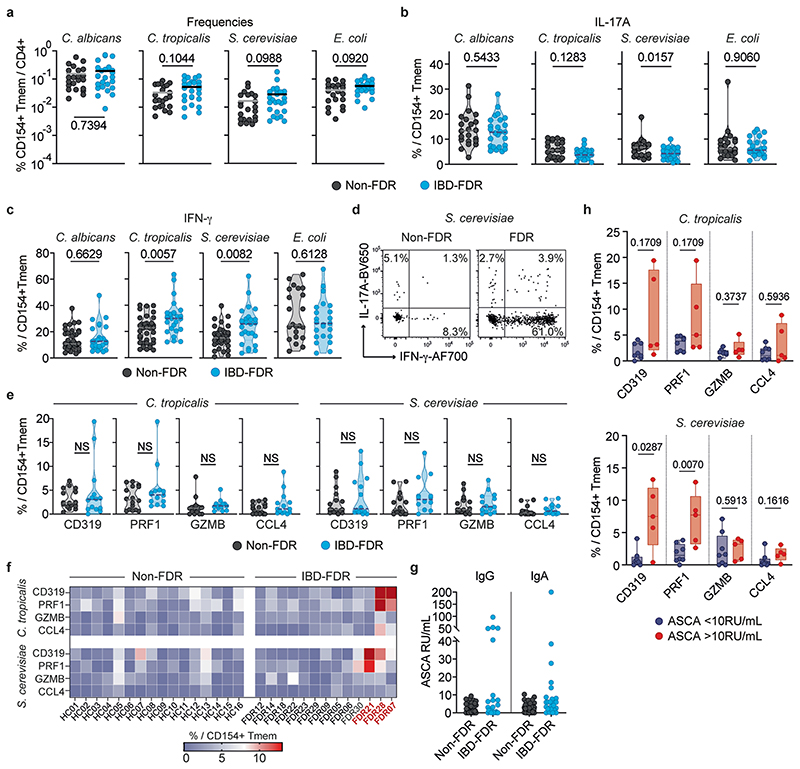
Alterations of yeast-reactive CD4+ T cells are present in first-degree-relatives of IBD patients (IBD-FDR). (**a**) Frequencies and (**b**) IL-17A production of yeast-reactive T cells in IBD-FDRs (n = 25) and non-FDR controls (n = 22). (**c**) IFN-γ staining of yeast-reactive T cells in IBD-FDRs (n = 25) and non-FDR healthy controls (n = 38). (**d**) Dot plot examples for IFN-γ staining of *S. cerevisiae*-reactive T cells. (**e**) Cytotoxic marker expression of *C. tropicalis* and *S. cerevisiae*-reactive CD154+ Tmem (non-FDR n = 16, IBD-FDR n = 14). (**f**) Heatmap depicting *ex vivo* cytotoxic markers production of yeast-reactive T cells within CD154+ Tmem measured by flow cytometry. (**g**) Serum anti-IgG and IgA ASCA antibody concentrations in non-FDRs (n = 46) and IBD-FDRs (n = 25). (**h**) Cytotoxic marker expression in IBD-FDRs in relation to elevated ASCA levels of the individual IBD-FDRs (ASCA < 10RU/ml n = 8, ASCA > 10 RU/ml n = 5). Individual donors are shown as dots. Each symbol in (**a**, **b**, **c**, **e**, **g**, **h**) represents one individual donor. Horizontal lines indicate mean values in (a). Truncated violin plots with quartiles and range are shown in (**b**, **c**, **e**). Box-and-whisker plots are shown in (**h**) with center lines represent the median, box limits represent 25% (lower) and 75% (upper) quartiles and whiskers represent minimum/maximum. Statistical differences: two-tailed Mann-Whitney test in (**a**, **b**, **c**, **e**, **h**).

So far, it remains unclear whether the development of yeast-responsive T_H_1-CTLs is a consequence of the disease or could eventually contribute to disease development and/or exacerbation. To address potential alterations of yeast-responsive T cells already in a predisease state, we analyzed a cohort of first-degree relatives of patients with IBD (IBD-FDRs), who have increased risk of developing IBD themselves^37,38^. We detected neither increased frequency nor increased IL-17A production of *C. tropicalis-* and *S. cerevisiae*-reactive T cells in IBD-FDRs compared with non-FDR controls (Extended Data Fig. 6a,b). However, *C. tropicalis-* and *S. cerevisiae*-reactive T cells from IBD-FDRs showed significantly increased IFN-γ production (Extended Data Fig. 6c,d), although less pronounced than for patients with CD (Fig. 2), whereas *Escherichia coli*-reactive cells, used as a control, were not altered (Extended Data Fig. 6c). In contrast to IFN-γ, cytotoxic markers were not significantly increased compared with non-FDR controls (Extended Data Fig. 6e). However, individual IBD-FDRs showed clearly elevated levels of CD319 and PRF1 on *C. tropicalis*- and/or *S. cerevisiae*-reactive T cells, whereas GZMB and CCL4 remained unchanged (Extended Data Fig. 6f). ASCA antibodies have been reported as a predictive biomarker that can be detected several years before the clinical diagnosis of CD^39,40^. Within our limited number of IBD-FDRs, we identified individuals with increased serum levels of ASCA IgG and IgA (Extended Data Fig. 6g). As for patients with CD, increased expression of cytotoxic markers was associated with elevated ASCA antibody titers in IBD-FDRs (Extended Data Fig. 6h).

In summary, these data indicate that a skewing toward a T_H_1 cell phenotype of yeast-responsive T cells is already detectable in IBD-FDRs and thus before the clinical onset of IBD. They further suggest that the additional acquisition of cytotoxic effector functions may evolve progressively, maybe as a result of a more intense or sustained interaction with yeast antigens, due to increased intestinal permeability, which has been described in IBD-FDRs^41^.

### Clonally expanded T_H_1-CTLs cross-react to multiple yeast species

**Fig. 5:**
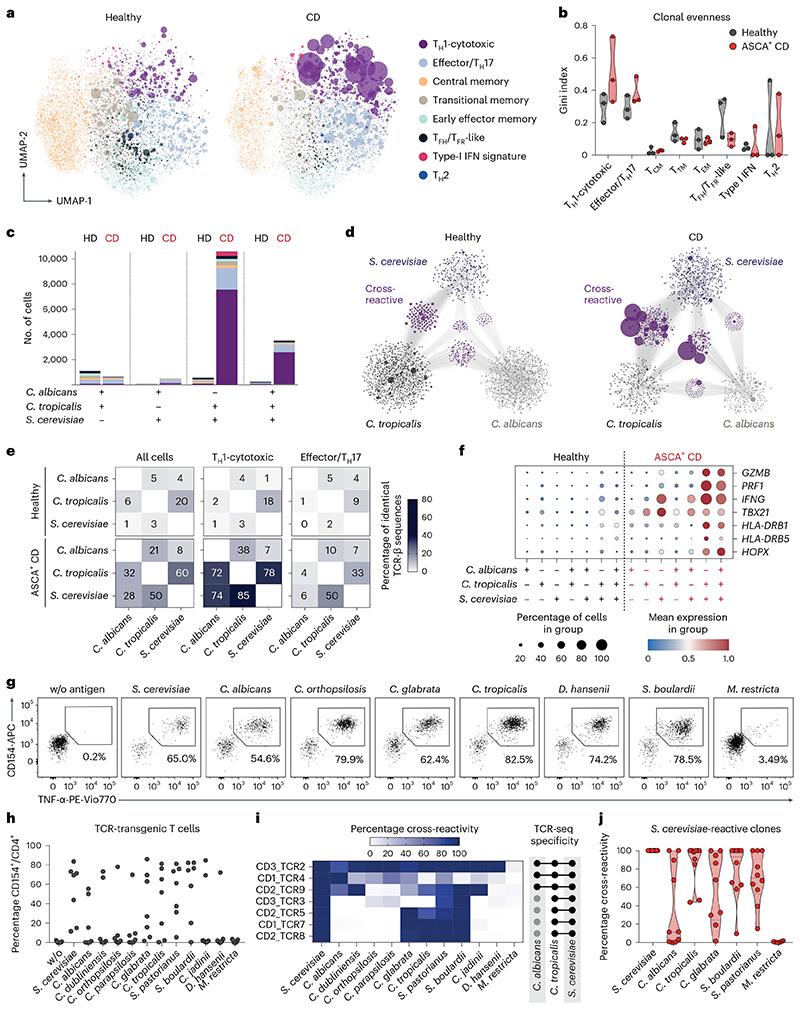
Clonally expanded T_H_1-CTLs are cross-reactive to multiple yeast species. **a**, TCR-β clonotype sizes were overlaid with UMAP visualization of the scRNA-seq dataset. **b**, Gini’s coefficient according to the different T cell clusters, depicting the distribution of TCR-β sequences (0 is total equality, that is, all clones have the same proportion; 1 is total inequality, that is, a population dominated by a single clone). To account for the uneven distribution of total sorted cells by samples, a down-sampled dataset was used taking 500 cells at random from each individual per fungal antigen (HD, *n* = 3; CD, *n* = 3). **c**, Number of T cells found in response to two or all three yeasts in healthy donors and patients with CD. The colors correspond to the different gene expression clusters from **a**. **d**, Clonal networks of antifungal TCR repertoires. Each dot represents a clonotype as defined by the TCR-β sequence. Connecting lines show TCR-β sequences that are shared between antifungal repertoires. The size of dots indicates clonal expansion. **e**, Percentage of identical TCR-β sequences found in samples stimulated with the different yeast species. **f**, Expression of selected marker genes according to TCR specificity. Colors represent the normalized mean expression levels and size indicates the proportion of cells expressing the respective genes. **g**, Cross-reactive TCRs from the single-cell dataset inserted into primary T cells using orthotopic TCR replacement. Dot plot examples show restimulation of expanded TCR-transgenic T cells for one TCR-α/β construct; numbers indicate percentage of reactive CD154^+^TNF^+^. **h**, Restimulation of TCR-transgenic T cells (*n* = 7) with different fungal species. **i**, Heatmap depicting the cross-reactivity of the individual TCR-transgenic T cells (*n* = 7) in relation to stimulation with *S. cerevisiae*. The initial observed crossreactivity based on TCR-seq is indicated on the right. **j**, *S. cerevisiae*-reactive, cytotoxic, CD154^+^CD319^+^, single-cell clones (*n* = 10) sorted ex vivo, expanded and restimulated with different fungal species. The percentage of cross-reactivity is indicated in relation to stimulation with *S. cerevisiae*. Each symbol in **b** represents one individual donor, in **h** one TCR-transgenic T cell line and in **j** one T cell clone. Truncated violin plots with quartiles and range are shown in **b** and **j**.

Our data so far suggest that chronic antigen stimulation may foster T_H_1-CTL development in patients with CD who are ASCA^+^. To better understand how chronic exposure to a large variety of commensal and dietary fungi may affect the clonal composition of yeast-reactive T cells, we integrated T cell receptor (TCR) sequencing data from our scRNA-seq dataset. Indeed, T_H_1-CTLs from patients with CD showed a massive expansion of individual clonotypes (Fig. 5a), further evidenced by a higher Gini’s coefficient, as a measure of the evenness of a population (Fig. 5b). Strikingly, many of the *S. cerevisiae*-stimulated TCRs of patients with CD were shared with *C. tropicalis-* and *C. albicans-*stimulated cells, whereas shared TCRs for the different fungi were less abundant in healthy donors (Fig. 5c,d). Indeed, most of the strongly expanded clonotypes were shared between the TCR repertoires reactive against two or all three fungi (Fig. 5d). TCR cross-reactivity was most pronounced within the T_H_1-CTL cluster, where >70% of the *S. cerevisiae*-reactive TCR-β sequences of patients with CD were also found in the *C. tropicalis-* and *C. albicans*-stimulated cells (Fig. 5e). This suggests that persistent or alternating exposure to conserved antigens present in different yeast species may promote the selective expansion of cross-reactive clonotypes. In particular, these cross-reactive clonotypes, but not clonotypes with a single species specificity, showed high expression of cytotoxic marker genes, as well as *HLA-DR* and *HOPX*, encoding for recent activation and chronic markers, respectively (Fig. 5f).

**Extended Data Fig. 7:**
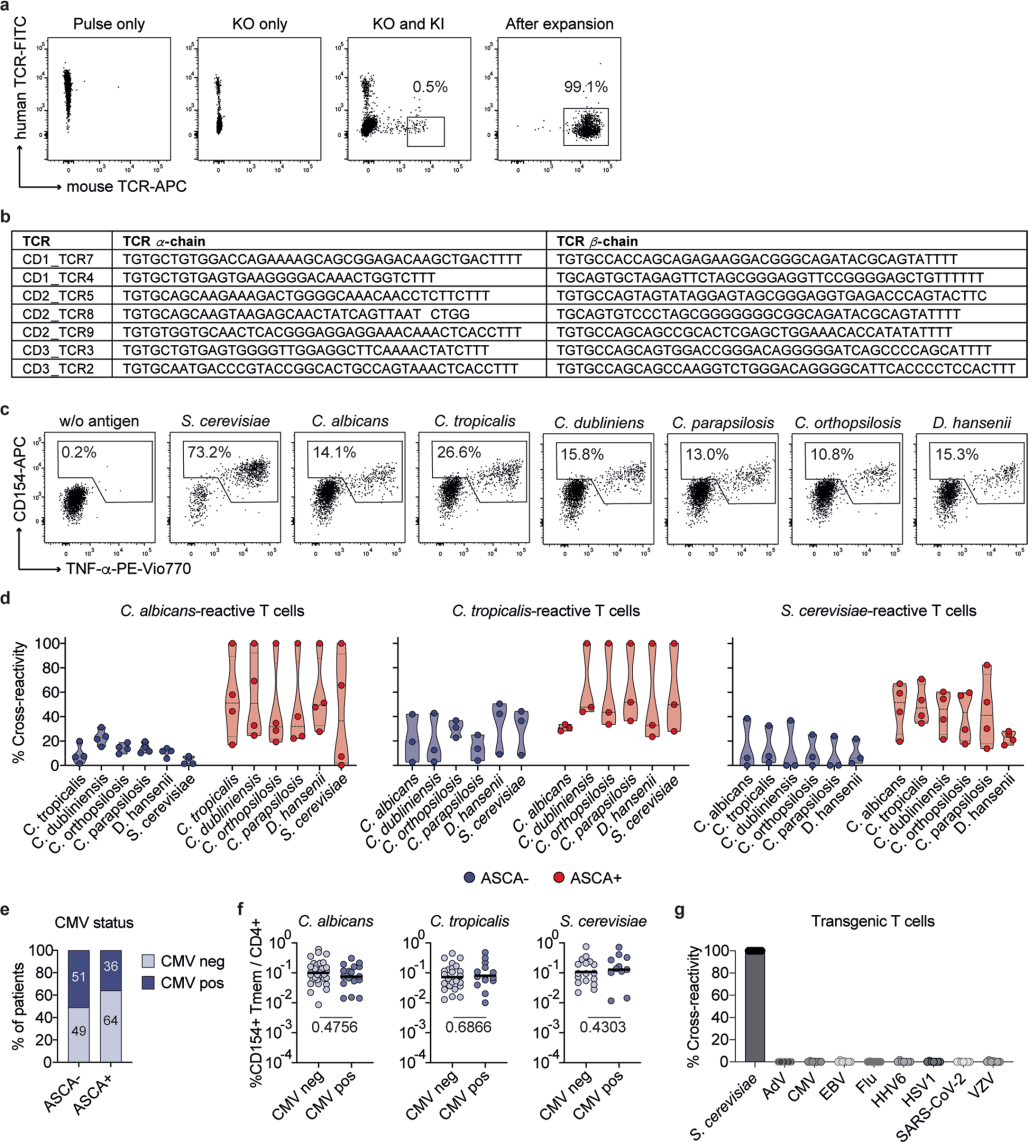
Cross-reactivity of yeast-responsive T cells. (**a**) α/β-TCR constructs of the most expanded cross-reactive TCRs identified from the single cell data set were inserted into primary human CD4+ T cells by orthotopic T cell receptor replacement using CRISPR/Cas. Dot plot examples showing CD4+ T cells with pulse only, knock-out of the endogenous TCR, combined knock-out of the endogenous TCR and knock-in of the transgenic TCR containing a murine β-chain constant region used as tracking marker. Transduced cells were further FACS purified and expanded to high purity. (**b**) TCR-α/β sequences selected from the scRNAseq data set for transgenic T cell generation. (**c**) *S. cerevisiae*-reactive Tmem from an ASCA+ CD patient were expanded and re-stimulated with various yeast species. Percentages of reactive CD154+ TNFα+ cells are indicated. (**d**) Cross-reactivity of expanded yeast-reactive T cells in ASCA- CD patients (*C. albicans* n = 4, *S. cerevisiae*, *C. tropicalis* n = 3) and ASCA+ CD patients (*C. albicans*, *S. cerevisiae* n = 4, *C. tropicalis* n = 3). Cross-reactivity is plotted as percentage in relation to total reactivity after stimulation with the initially used yeast species. (**e**) CMV serum antibody status of ASCA- CD patients (n = 39) and ASCA+ CD patients (n = 49) was determined by ELISA and percentage of CMV negative and CMV positive patients is indicated. (**f**) Yeast-reactive T cell frequencies of ASCA+ patients according to their CMV status (*C. albicans* CMVneg n = 32, CMVpos n = 17; C. tropicalis CMVneg n = 24, CMVpos n = 14; *S. cerevisiae* CMVneg n = 17, CMVpos n = 11). (**g**) TCR transgenic T cells (n = 7) were restimulated with common viral antigens. Cross-reactivity in relation to stimulation with *S. cerevisiae* is shown. Each symbol in (**d**, **f**) represents one individual donor and in (**g**) one TCR transgenic T cell line. Truncated violin plots with quartiles and range are shown in (**d**). Horizontal lines indicate geometric mean values in (**f**). Statistical differences: two-tailed Mann-Whitney test in (**f**).

To further confirm the T cell cross-reactivity for individual TCRs, we re-expressed the most expanded cross-reactive T_H_1-CTL TCRs from the single-cell dataset in primary T cells using clustered regularly interspaced palindromic repeats (CRISPR)–Cas9^42,43^ (Extended Data Fig. 7a,b). The expanded TCR-transgenic T cells were re-challenged with a panel of different yeasts in the presence of autologous antigen-presenting cells (APCs). As shown in Fig. 5g–i, we observed high cross-reactivity of the transgenic T cells to several yeast species and the reactivity of individual TCRs further reflected the cross-reactivity pattern initially observed by TCR-seq (Fig. 5i). High cross-reactivity to several yeast species was also confirmed for ex vivo sorted and expanded, *S. cerevisiae-*reactive, cytotoxic, CD154^+^CD319^+^ single-cell clones (Fig. 5j). Finally, to validate the selective enrichment of cross-reactive cells in patients who were ASCA^+^, we expanded bulk *C. albicans-, C. tropicalis-* and *S. cerevisiae*-reactive CD154^+^ T_mem_ cells from patients with CD. Yeast-reactive T cells derived from patients with CD who are ASCA^+^, but not ASCA^−^, highly cross-reacted to various yeast species (Extended Data Fig. 7c,d), consistent with the presence of these cells in patients who are ASCA^+^. Cytotoxic CD4^+^ T cells have so far been mainly described in the context of persistent viral infections^32^. However, none of the TCR-transgenic T cells reacted to common viral antigens and we also observed no differences between patients with CD who are cytomegalovirus (CMV) positive and those who are CMV negative in the frequencies of yeast-responsive T cells (Extended Data Fig. 7e–g).

Taken together, these data show that CD4^+^ T_H_1-CTLs that cross-recognize various yeast species are strongly expanded in patients with CD who are ASCA^+^. The repetitive encounter with conserved antigens, present in different yeast species, may thus lead to the selective expansion of the cross-reactive clones, which progress to cytotoxic cells owing to chronic stimulation in patients with CD.

### CD4^+^ T_H_1-CTLs respond to food-derived yeast antigens

**Fig. 6:**
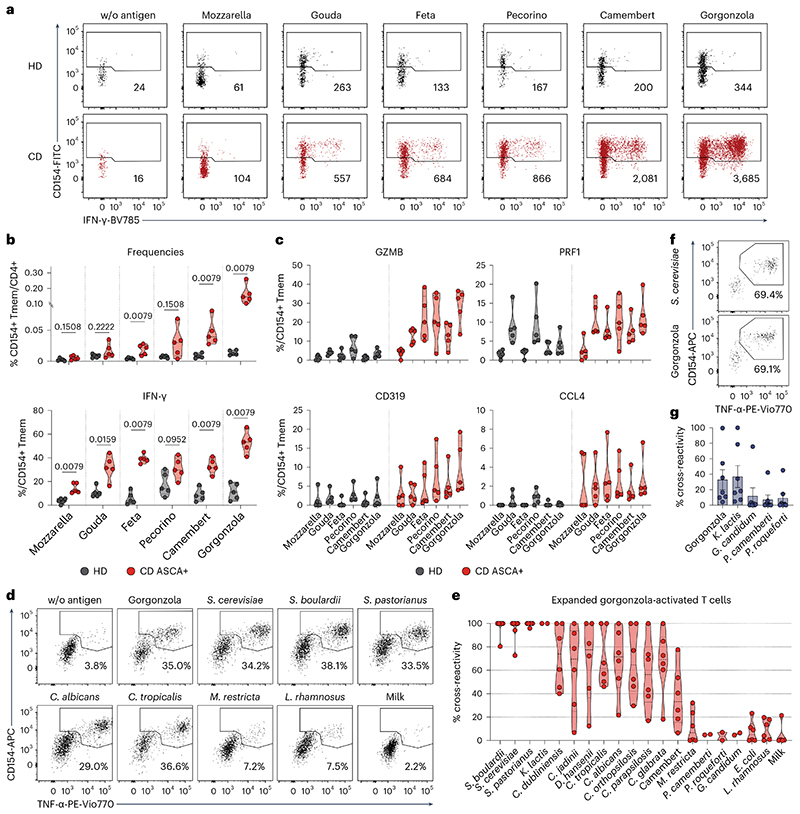
Food-derived yeasts can activate cross-reactive T_H_1-CTLs in patients with CD. **a**–**c**, PBMCs of healthy donors and patients with CD were stimulated with lysates of different cheese types and reactive CD4^+^ T cells analyzed by ARTE. **a**, Dot plot examples. Absolute cell counts after magnetic CD154^+^ enrichment from 1 × 10^7^ PBMCs are indicated. **b**, CD154^+^ T_mem_ cell frequencies and IFN-γ production in response to different cheese types (HD, *n* = 5; ASCA^+^ CD, *n* = 5). **c**, Ex vivo cytotoxic marker expression of reactive CD154^+^ T_mem_ cells in response to different cheese types (HD, *n* = 5; ASCA+ CD, *n* = 5). **d**, Reactive CD154^+^ T_mem_ cells from patients with CD who are ASCA^+^ were isolated after stimulation with gorgonzola lysate and expanded. Dot plots show reactivity on restimulation with the indicated antigens. **e**, Cross-reactivity of expanded gorgonzola-activated cells from patients with CD who are ASCA^+^ to different microbial and control antigens (*Kluyveromyces lactis*, *Penicllium camemberti*, *P. roqueforti*, *Geotrichum candidum*, *n* = 2; all other antigens, *n* = 6). **f**,**g**, TCR-transgenic T cells restimulated with lysates of gorgonzola or different fungal species commonly found in cheese. **f**, Dot plot examples with percentage of reactive CD154^+^TNF^+^ cells shown for one TCR-α/β construct. **g**, Crossreactivity of the individual TCR-transgenic T cells (*n* = 7) in relation to stimulation with *S. cerevisiae*. Each symbol in **b**, **c** and **e** represents one individual donor and in **g** one TCR-transgenic T cell line. Truncated violin plots with quartiles and range are shown in **b**, **c** and **e** and mean values with s.e.m. in **g**. Statistical differences were obtained using the two-tailed Mann–Whitney *U*-test in **b**.

The finding of a substantial cross-recognition of various yeast species by the CD4^+^ T_H_1-CTLs prompted us to investigate potential further antigenic triggers of these cells. As several yeast species are widespread organisms and frequently consumed with the diet, we hypothesized that food-derived yeast species could also promote the activation of CD4^+^ T_H_1-CTLs from patients with CD. Cheese contains live microorganisms, including various *Candida, Saccharomyces, Penicillium* and several other fungal species^44,45^. To analyze whether food-derived fungal species could activate CD4^+^ T_H_1-CTLs in patients with CD, we stimulated PBMCs with heat-inactivated lysates of common cheese types and analyzed the reactive T cells by ARTE. Patients with CD who are ASCA^+^, but not healthy donors, showed clearly increased reactivity, especially to the mold-ripened cheeses camembert and gorgonzola (Fig. 6a,b). In line with our previous results, these cheese-activated T cells from patients with CD produced increased amounts of IFN-γ (Fig. 6b) and expressed elevated levels of cytotoxic markers (Fig. 6c). To confirm that the T cell reactivity observed with the cheese lysates was the result of T cell cross-reactivity against yeasts, we expanded gorgonzola-activated CD154^+^ T_mem_ cells and restimulated them with different microbial species. Indeed, expanded T cell lines from patients with CD who are ASCA^+^ exhibited up to 100% cross-reactivity to several yeast species, but not bacteria or milk proteins (Fig. 6d,e), indicating that the observed reactivity against the cheese is probably directed against the yeast species present in it. This was further confirmed by reactivity of the TCR-transgenic cells against the gorgonzola lysate (Fig. 6f,g), as well as against the yeast *Kluyveromyces lactis* (formerly *Saccharomyces lactis*), whereas reactivity to phylogenetic distant *Penicillium* or *Geotrichum* mold species, which are also frequently contained in cheese^44,45^, was low.

In summary, our data reveal that, in addition to gut-resident commensals, food-derived yeasts can also activate the cytotoxic T_H_1-CTLs in patients with CD. The selective expansion of cross-reactive CD4^+^ T cells by different members of the mycobiota, as identified in the present study, may contribute to chronicity and treatment resistance of CD, when persistently activated by different commensal and food-derived fungi.

## Discussion

An inappropriate immune response against intestinal microorganisms has been suggested as a major contributor to the pathophysiology of CD. Despite extensive data supporting the involvement of T cells in IBD pathogenesis, the relevant intestinal antigens and the effector functions of the corresponding antigen-specific T cells, remain largely unknown. In the present study, we provide direct evidence that common intestinal fungal commensals, as well as food-derived yeasts, contribute to aberrant inflammatory CD4^+^ T cell responses in patients with CD.

We observed massively increased CD4^+^ T cell responses against different yeast species in patients with CD. In addition to *C. albicans*, which has been identified as a strong modulator of human adaptive immunity^15–17,22,46^, the altered T cell response against *non-albicans Candida* and food-associated *Saccharomyces* spp. adds an additional layer to the inflammatory immune reaction in CD. Development of pathogenic T_H_17 cells has been discussed as contributing to IBD pathology and we recently showed that *C. albicans* is the major driver of fungus-induced homeostatic T_H_17 cell responses in humans^15^. In the present study, we observed that the proportion of T_H_17 cells against *C. albicans* was surprisingly unaffected in patients with CD, but that cytotoxic T_H_1 cells develop that also strongly react against *non-albicans Candida* spp., as well as different *Saccharomyces* yeasts. Which fungal species is the initial trigger of the T_H_1 response and which fungi merely drive the selection of cross-reactive TCR remains unresolved. Recently, *Debaryomyces hansenii*, a yeast commonly contained in dairy products, has been described as being specifically enriched in inflamed wounds of patients with CD^18^, suggesting that food-derived fungi may also adapt to the altered environment in the IBD gut. In this way, both intestinal fungal commensals and food-derived species could be involved in the initial priming of the T_H_1 cell response observed here. Once established, these T_H_1 cells may be propagated by multiple fungal species, leading to the evolution of a highly selected cross-reactive TCR repertoire.

The limited impact of IBD on the T_H_17 cell reaction against *C. albicans* suggests that the strong homeostatic T_H_17 cell response present in most individuals^15^ is relatively inert to perturbations. Why T_H_17 cell responses against *C. albicans* are less affected by IBD remains unclear. It is possible that fungus-reactive T_H_1 and T_H_17 cells recognize different antigens that have different availability in CD than in healthy individuals. Indeed, we detected high TCR cross-reactivity within the yeast-responsive T_H_1-CTLs, compared with T_H_17 cells. This indicates that T_H_1-CTLs, but not T_H_17 cells, are directed against conserved epitopes that are commonly present in many yeast species, thereby also increasing antigen abundance. Alternatively, a conversion of T_H_17 into T_H_1 cells may occur on continuous antigen stimulation in chronic inflammation^5,34^. However, yeast-reactive T_H_17 and T_H_1 cell cytokines were mainly produced by different cell populations and only small populations of IL-17A^+^FN-γ^+^ cells develop against *non-albicans Candida* and *Saccharomyces* spp. Altogether this suggests that T_H_17 cells and T_H_-CTLs represent two independent populations driven most probably by separated groups of antigens. In future studies, it will be important to define specific fungal proteins and peptides that are recognized by T_H_17 cells and cross-reactive T_H_-CTLs, which may allow the development of targeted, antigen-specific, therapeutic modulations. The cross-recognized antigens could comprise precisely the same or similar epitopes present in different yeast species or even unrelated sequences forming structurally similar epitopes, which moreover could also vary for different TCRs.

Overall, our data suggest a model in which, after initial T_H_1 cell priming, repeated exposure to conserved antigens present in multiple fungal species leads to the selection of a highly cross-reactive TCR repertoire, accompanied by the acquisition of cytotoxic effector functions, probably as a result of chronic T cell activation. This is also supported by the observation that increased IFN-γ production and certain cytotoxic markers were elevated on yeast-responsive T cells in individual IBD-FDRs, but not yet the entire cytotoxic phenotype. Furthermore, the most expanded cross-reactive clonotypes from patients with CD showed the highest gene expression of cytotoxic markers, as well as the chronic activation marker gene *HOPX*^33,34^. As healthy individuals also harbor commensal intestinal yeasts and frequently ingest *S. cerevisiae* in the diet, the observed altered T cell reactivity in patients with CD and the incipient changes in individual IBD-FDRs may reflect a more intense or sustained interaction with yeast antigens, owing to epithelial barrier dysfunction. T cell-mediated cytotoxicity has been described as contributing to IEC death^47,48^ and exacerbated T_H_1 immunity in mice promoted epithelial barrier defects and mucosal fungal infection^49^. It is therefore tempting to speculate that yeast-responsive T_H_-CTLs may be directly involved in disease initiation and/or exacerbation through disruption of the intestinal barrier. In this regard, cross-reactive, yeast-responsive TCRs could be prime targets for the development of specific therapeutic interventions by antigen-specific regulatory T cell (T_reg_ cell) therapy or T cell-depleting strategies.

Yeast-reactive T cells were selectively altered in patients with CD who are ASCA^+^, but not in those who are ASCA^−^ or in patients with UC. It is thus possible that yeast-reactive T_H_-CTLs or their T_H_1 progenitors may be involved in providing B cell help for ASCA formation. The presence of ASCAs in CD was described more than 30 years ago^50,51^ and ASCAs are used as a biomarker for disease severity^30^, as well as a prognostic marker in individuals at risk for developing IBD^40^. Despite this strong link, their direct pathogenic relevance has been questioned and the yeast-responsive T cells have so far not been analyzed in detail in patients with CD. *S. cerevisiae*, also known as bakers’ yeast, is ingested daily via nutritional intake and therefore generally considered to have a low virulence in humans. Instead, *C. albicans*, due to its higher immunogenicity, has been speculated to be the inducing species, as a result of binding of ASCAs to *C. albicans* mannans^24^. Given the data presented in the present study, it will be important to further define the fungal species and mechanisms involved in ASCA formation and in particular the contribution of T_H_1-CTLs.

Our data may also result in concepts for selecting patients with CD for particular therapies. For example, therapeutic inhibition of Janus kinases targets, among other cytokines, the release of IFN-γ and might show more effectivity in patients with CD who are ASCA^+^. Further studies will examine the role of nutritional interventions with selective removal of yeast antigens, which—if effective—would be an attractive treatment strategy supporting the efficacy of other anti-inflammatory therapies or remission maintenance. Antifungal therapy, as well as a yeast-free diet, will be analyzed in future clinical trials in patients with CD who are ASCA^+^.

Our study also has some limitations. Although we have provided strong correlative evidence for the involvement of yeast-reactive CD4^+^ T cells in CD, further investigations are necessary to determine their direct contribution to disease pathophysiology. Our initial attempts in this direction provide some encouraging results and evidence for the ability of these cells to directly kill intestinal epithelium in vitro. However, further studies need to clarify whether therapeutic targeting of intestinal yeasts or yeast-responsive T_H_1-CTLs is clinically effective. Although these highly cross-reactive T cells in patients with CD react to multiple yeast species, future studies are needed to determine whether a single or multiple species serve as the initial trigger for the altered yeast-reactive T cell response. In the present study, we analyzed only a limited number of bacterial and fungal species and potential differences induced by strain variation were not addressed. Furthermore, the proteins or peptides driving the cross-reactive, yeast-responsive population remain to be determined. The discovery that a specific subgroup of patients with CD develops altered CD4^+^ T cell responses and ASCAs against yeasts, whereas other patients with IBD do not, underscores the presence of fundamentally distinct host–microbe interactions within this subgroup of patients. This emphasizes the need for in-depth investigations to shed light on the underlying mechanisms and to discern the unique characteristics of this subgroup of patients with CD.

## Online content

Any methods, additional references, Nature Portfolio reporting summaries, source data, extended data, supplementary information, acknowledgements, peer review information; details of author contributions and competing interests; and statements of data and code availability are available at https://doi.org/10.1038/s41591-023-02556-5.

## Methods

### Blood donors

Peripheral EDTA blood samples of healthy donors were obtained from blood bank donors of the Institute for Transfusion Medicine, UKSH Kiel, Germany or from in-house volunteers (ethics committee CAU Kiel D578/18, D427/19). Peripheral EDTA blood samples from patients with IBDs were obtained from the Department of Internal Medicine I, UKSH Kiel, the Comprehensive Center for Inflammation Medicine and the Gastroenterology–Hepatology Center Kiel (ethics committee CAU Kiel D427/19). IBD-FDRs were recruited from the Kiel IBD Family Cohort Study, a German-wide cohort of patients with IBD and their family members (ethics committee CAU Kiel A117/13), built by the popgen Biobank at Kiel University/University Hospital SH in Kiel. Demographic data about the study participants are provided in Extended Data Tables 1 and 2. CMV serostatus was determined using an anti-CMV IgG ELISA according to the manufacturer’s instructions (Serion Diagnostics). All blood donors gave written informed consent before participation.

### Intestinal biopsies

Biopsies from inflamed and noninflamed intestinal tissue were collected from patients with CD who underwent colonoscopy (ethics committee CAU Kiel, under the file: B 231/89-1/13). All patients gave written informed consent before participation.

### Small IECs

Primary small IECs were purchased from PELOBiotech. Cells were cultivated in Epithelial Cell Medium (Cell Biologics) in T25 flasks pretreated with gelatin-based coating solution (Cell Biologics). During expansion, medium was replenished and cells were split as needed.

### Quantification of ASCAs

Serum ASCAs were measured by ELISA according to the manufacturer’s instructions (Euroimmun). In short, ASCA IgA/IgG ELISA kits include three calibration sera, a positive and a negative control. Sera were diluted 1:101 in probe buffer and incubated at room temperature on precoated microtiter plates for 30 min. After washing, a peroxidase-conjugated, anti-human IgA or IgG was added for 30 min and the plates were washed again. Substrate solution was added and the color reaction was stopped after 15 min. The optical density was determined at 450 nm, with a reference wavelength of 620 nm. ASCA status was calculated after developing a standard curve using the three calibration sera. ASCA quantification was done in duplicate and mean values were used. Sera were considered positive if their activity was >20 relative units ml^−1^. Both single-positive IgA or IgG samples and double-positive samples were classified as ASCA^+^ donors.

### ITS-sequencing

ITS (internal transcribed spacer) amplicons were generated with 35 cycles using Invitrogen AccuPrime PCR reagents. These amplicons were used in a second PCR reaction, applying Illumina Nextera XT v.2 (Illumina) barcoded primers to uniquely index each sample as previously described^20^. All libraries were processed for quality control using the DNA 1000 Bioanalyzer (Agilent) and Qubit (Life Technologies) to validate and quantify library construction before preparing a paired-end flow cell. The samples were randomly divided among flow cells to minimize sequencing bias. Clonal bridge amplification (Illumina) was performed using a cBot (Illumina); 2× 250-base pair (bp) sequencing-by-synthesis was performed using the Illumina MiSeq platform.

### Generation of microbial antigen lysates

Bacterial and fungal lysates were generated according to previously described methods^15,52^. In brief, bacteria *E. coli* (DSM 8698), *Faecalibacterium prausnitzii* (DSM 17677), *Bacteroides fragilis* (American Type Culture Collection (ATCC) 25285), *Clostridium leptum* (DSM 753), *Prevotella copri* (DSM 18205), *Akkermansia muciniphila* (DSM 22959) and *Klebsiella pneumoniae* (clinical isolate) were grown on solid medium (Columbia Blood Agar, Biomérieux), harvested by scraping into 17.4 ml of distilled water and lysed by addition of 0.4 ml of NaOH (1 M), 0.2 ml of HCl (2 M) and 2 ml of 10× phosphate-buffered saline (PBS), pH 7.5. Lysis of cells was confirmed by microscopic analysis and by negative culture of lysates. Heat-killed *Lactobacillus rhamnosus* was purchased from Invivogen.

Lyophilized extract of *C. albicans* (SN-1 6946) was purchased from Greer Laboratories. *S. cerevisiae* (ATCC 9763)*, S. boulardii* (Perenterol), *S. pastorianus* (DSM 6580), *Cyberlindnera jadinii* (CBS 1600), *C. dubliniensis* (CD36), *C. tropicalis* (DSM 11953), *Candida glabrata* (ATCC 200l), *C. parapsilosis* (GA1), *C. orthopsilosis* (ATCC 96141), *Debaryomyces hansenii* (JMRC STI25082), *K. lactis* (formerly *S. lactis*; STH00086) and *G. candidum* (SF012383) were first grown on yeast-peptone-dextrose (YPD) agar plates (10 g l^−1^ of peptone, 5 g l^−1^ of yeast extract, 20 g l^−1^ of dextrose and 15 g l^−1^ of agar). For protein extraction, liquid cultures of *S. pastorianus* and *C. jadinii* were grown in synthetic defined (SD) medium (2% glucose, 6.7 g l^−1^ of yeast nitrogen base without amino acids, 20 mg l^−1^ of histidine,120 mg l^−1^ of leucine, 60 mg l^−1^ of lysine, 20 mg l^−1^ of arginine, 20 mg l^−1^ of tryptophan, 20 mg l^−1^ of tyrosine, 40 mg l^−1^ of threonine, 20 mg l^−1^ of methionine, 50 mg l^−1^ of phenylalanine, 20 mg l^−1^ of uracil and 20 mg l^−1^ of adenine), *K. lactis* and *G. candidum* in SD + CSM (2% glucose, 6.7 g l^−1^ of yeast nitrogen base without amino acids, 1× complete supplement mixtures (CSM, Formedium)), *D. hansenii* in liquid Sabouraud 2% (w/v) glucose-bouillon (Carl Roth), and *C. dubliniensis, C. tropicalis, C. glabrata, C. parapsilosis* and *C. orthopsilosis* in liquid YPD medium overnight at 30°C. *M. restricta* (CBS-7877) and *M. furfur* (DSM 6170) were cultured in 250 ml of MLNA medium (10 g l^−1^ of bacteriological peptone, 10 g l^−1^ of glucose, 2 g l^−1^ of yeast extract, 8 g l^−1^ of desiccated ox bile, 10 ml l^−1^ of glycerol, 0.5 g l^−1^ of glycerol monostearate, 5 ml l^−1^ of Tween-80 and 20 ml l^−1^ of olive oil) at 32°C for 4 d.

Yeast cells were harvested by centrifugation for 5 min at ≥1,500*g* and washed 3× to remove culture medium. Cell pellets were disrupted in a tissue homogenizer with glass beads for at least three rounds at high intensity, with 5 min of incubation on ice in between. All microbial extracts were centrifuged at 15,000–20,000*g* for 1–15 min to remove debris, supernatants were passed through a 0.2-μm sterile filter (Sartorius, Minisart) and stored in aliquots at -70 °C until use.

*Penicillium roqueforti* (SF006507) and *P. camemberti* (STN02007) were cultivated at 20°C for 7–10 d on malt agar plates and germinated in *Aspergillus* minimal medium with glucose as the sole carbon source at 24 °C until sufficient mycelial pellets were present. Mycelia were separated from the medium with Miracloth (Merck Millipore), washed with water and protein extracts were generated by breaking the mycelium in liquid nitrogen by grinding using a mortar and pestle.

To generate suspensions from cheese, cheeses were cut into small pieces and put into gentleMACS M-tubes (Miltenyi Biotec) containing 3 ml of 1× PBS buffer. Cheeses were disrupted using the gentleMACS device (Miltenyi Biotec) program RNA.01 3×. Tubes were centrifuged at 1,000*g* for 10 min and supernatants were heat inactivated for 10 min at 65 °C.

All microbial lysates were used in at a protein concentration of 40 μg ml^−1^ for stimulation and cheese suspensions were used at a protein concentration of 200 μg ml^−1^.

### Other antigens

The following control antigens were used for stimulation: lysates of CMV (Siemens Healthcare Diagnostics), adenovirus (AdV; Microbix), varicella-zoster virus (Microbix) and Epstein–Barr virus (Hölzel Biotech). All viral lysates were used at a concentration of 10 μg ml^−1^ for stimulation. Peptide pools of herpes simplex virus 1 (Envelope Glycoprotein D; Miltenyi Biotec), influenza A H1N1 (pool of HA, MP1, MP2, NP, NA; Miltenyi Biotec), human herpesvirus 6 (pool of U54, U90; peptides&elephants) and SARS-CoV-2 (spike protein; JPT). Peptide pools were resuspended according to manufacturers’ instructions and cells were stimulated at a concentration of 0.5 μg per peptide per ml. Cows’ milk protein extract was purchased from Greer Laboratories and used at a concentration of 100 μg ml^−1^.

### Antigen-reactive T cell enrichment

PBMCs were freshly isolated from EDTA blood on the day of blood donation by density gradient centrifugation (Biocoll, Biochrom). ARTE was performed as previously described^15,28,52–54^, with slight modifications. In brief, 1–2 × 10^7^ PBMCs were plated in RPMI-1640 medium (Gibco), supplemented with 5% (v/v) human AB-serum (Sigma-Aldrich) in 12-well cell-culture plates and stimulated for 7 h with microbial lysates in the presence of 1 μg ml^−1^ of CD40 and 1 μg ml^−1^ of CD28 pure antibody (both Miltenyi Biotec). Then, 1 μg ml^−1^ of Brefeldin A (Sigma-Aldrich) was added for the last 2 h. In some experiments 100 μg ml^−1^ of anti-HLA-DR pure antibody (Miltenyi Biotec; clone AC122 or BioXcell; clone LT43) was added during stimulation. To multiplex several specificities, the differentially stimulated cells were labeled with different concentrations of two CD4 antibody clones (CD4-BV421, BioLegend, clone OKT4, titer 1:20 and 1:200; CD4-APC-Vio770, Miltenyi Biotec, clone MT-466, titers 1:50 and 1:500). For lower concentrations, the respective unconjugated CD4 pure antibody was added at a concentration of 1 μg ml^−1^ to block intermixing of the barcode label. Barcoded populations were pooled and labeled with CD154-biotin followed by anti-biotin MicroBeads (CD154 MicroBead Kit, Miltenyi Biotec) and magnetically enriched by two sequential MS Columns (Miltenyi Biotec). Surface staining was performed on the first column, followed by fixation and intracellular staining on the second column. Frequencies of antigen-specific T cells were determined based on the cell count of CD154^+^ T cells after enrichment, normalized to the total number of CD4^+^ T cells applied on the column. For each stimulation, CD154^+^ background cells enriched from the nonstimulated control were subtracted.

### Flow cytometry

Cells were stained in different combinations of fluorochrome-conjugated antibodies (clone names and titers in parentheses): CD3-PE (REA613), CD4-APC-Vio770 (M-T466), CD4-VioBlue (REA623), CD4-FITC (REA623), CD8-VioGreen (REA734), CD8-PerCP (BW135/80), CD14-VioGreen (REA599), CD14-PerCP (TÜK4), CD20-VioGreen (LT20), CD20-PerCP (LT20), CD45RA-VioGreen (REA562), CD45RO-APC (REA611), CD69-PE (REA824), CD154-FITC (REA238), CD154-APC (REA238), TNF (tumor necrosis factor)-PE (phycoerythrin)-Vio770 (cA2), CD197 (CCR7)-PE-Bio770 (REA108), IL-17A-PE-Vio770 (REA1063), IL-21-VioR667 (REA1039), IL-21-PE (REA1039), perforin-PE (REA1061), GM-CSF-APC (REA1215), GM-CSF-PE-Vio770 (REA1215) (all Miltenyi Biotec, all in titer 1:50), CD4-BV421 (OKT4, 1:20), CD45RA-PE-Cy5 (HI100, 1:60), IFN-γ-BV785 (4 S.B3, 1:20), IL-2-BV605 (MQ1-17H12, 1:20), IL-2-BV711 (MQ1-17H12, 1:50), IL-10-PE-Dazzle-594 (JES3-9D7, 1:50), IL-17A-BV605 (BL168, 1:20), Granzyme-B-PerCP-Cy5 (QA16A02, 1:20), CD319-PE-Dazzle-594 (162.1, 1:20), TNF-BV650 (MAb11, 1:20), anti-mouse-TCR-β-APC (H57-597, 1:20), anti-human-TCR-αβ-FITC (IP26, 1:20) (all BioLegend), and IL-17A-BV650 (N49-653, 1:20), integrin b7-BV650 (FIB504, 1:20), CCL4 (MIP-1b)-AF770-(D21-1351, 1:20), Ki-67-AF700 (B56, 1:20), Ki-67-BV480 (B56, 1:20) and IL-22-PerCP-eFluor710 (IL22JOP, 1:100) (all BD Biosciences). Viability 405/520 Fixable Dye 1:100 (Miltenyi Biotec) was used to exclude dead cells. For intracellular staining, cells were fixed and permeabilized with the inside stain kit (Miltenyi Biotec). Data were acquired on an LSR Fortessa (BD Bioscience) or Northern Lights 3000 (Cytek Biosciences). Screening of expanded T cell lines on 384-well plates was performed on a MACSQuantX Analyzer (Miltenyi Biotec). FlowJo v.10.9.0 (Tree Star) software was used for analysis.

### Expansion and restimulation of antigen-reactive T cells

For expansion of antigen-specific T cells, PBMCs were stimulated for 6 h in the presence of 1 μg ml^−1^ of CD40 and 1 μg ml^−1^ of CD28 pure antibody, CD154^+^ cells were isolated by magnetic activated cell sorting (MACS) and further purified by FACS on a FACS Aria Fusion (BD Bioscience) based on CD154^+^CD69^+^CD45RA^−^ expression into 96-well, round-bottomed plates. For generation of yeast-responsive T_H_1-CTL single-cell clones, *S. cerevisiae*-stimulated CD154^+^CD69^+^C D45RA^−^CD319^+^ cells were sorted by FACS as single cells into 96-well, round-bottomed plates.

Bulk or single-cell sorted T cells were expanded in the presence of 1 × 10^5^ autologous, antigen-loaded irradiated feeder cells (negative fraction from CD154^+^ MACS) in TexMACS medium (Miltenyi Biotec), supplemented with 5% (v/v) human AB-serum (GemCell), 200 U ml^−1^ of IL-2 (Proleukin; Novartis), 100 IU ml^−1^ of penicillin, 100 μg ml^−1^ of streptomycin, 0.25 μg ml^−1^ of amphotericin B (antibiotic antimycotic solution, Sigma-Aldrich), 20 μM 2-mercaptoethanol (Gibco, Life Technologies), 2 mM glutamine and 30 ng ml^−1^ of anti-CD3 (OKT-3; Miltenyi Biotec). After 7 d, 100 ml of the medium was replenished and 1 × 10^5^ irradiated feeder cells were added. During expansion for 2–4 weeks, medium was replenished and cells were split as needed.

For restimulation, fastDCs were generated from autologous CD14^+^ MACS-isolated monocytes (CD14 MicroBeads; Miltenyi Biotec) by cultivation in X-Vivo15 medium (BioWhittaker/Lonza), supplemented with 1,000 IU ml^−1^ of GM-CSF and 400 IU ml^−1^ of IL-4 (both Miltenyi Biotec). Before restimulation, expanded T cells were rested in RPMI-1640 supplemented with 5% human AB-serum without IL-2 for 2 d. Then, 0.5–1 × 10^5^ expanded T cells were plated with fastDCs in a ratio of 1:1 of in 384-well, flat-bottomed plates and restimulated for 6 h, with 1 mg ml^−1^ of Brefeldin A added for the last 4 h. Cells were stained intracellularly for CD154 and cytokines.

### Expansion of CD4^+^ T cells from intestinal biopsies

Up to 4 biopsies per patient were pooled and digested in 1 mL of Hanks’ balanced salt solution Ca^2+^Mg^2+^ (Gibco) supplemented with 0.5% (v/v) human AB-serum, 100 IU ml^−1^ of penicillin, 100 μg ml^−1^ of streptomycin, 0.25 μg ml^−1^ of amphotericin B, 10 IU ml^−1^ of DNase I (CellSystems) and 1 mg ml^−1^ of collagenase type CLS IV (Biochrom) for 30 min at 37 °C while shaking. Cells were filtered through a 40-μm cell strainer to remove aggregates and stained with fluorochrome-conjugated antibodies to CD4-VioBlue, CD3-PE, CD8-PerCP, CD14-PerCP, CD20-PerCP, CD45RO-APC and CCR7-PE-Vio770 (all Miltenyi Biotec). Each 200 CD3^+^CD4^+^CD45RO^+^ cells were sorted in multiple wells of a 96-well plate containing irradiated allogeneic feeder cells in TexMACS medium, supplemented with 5% (v/v) human AB-serum, 200 U ml^−1^ of IL-2 and 100 IU ml^−1^ of penicillin, 100 μg ml^−1^ of streptomycin, 0.25 μg ml^−1^ of amphotericin B at a density of 2 × 10^5^ cells cm^−2^. Cells were polyclonally expanded for 4 weeks in the presence of 30 ng ml^−1^ of anti-CD3 (OKT-3; Miltenyi Biotec). Restimulation with different fungal lysates was performed as described above. To calculate the frequencies of reactive T cells, the mean values were calculated for each antigen and divided by the number of input wells.

### Cytotoxicity assay

Cytotoxicity of yeast-reactive CD4^+^ T cells against adherent small IECs (PELOBiotech) was measured in duplicate by a Real Time Cell Analyzer (RTCA, X-Celligence, ACEA)^55^. By using RTCA, the impedance of the cells is monitored via electronic sensors located on the bottom of a 96-well micro-E-plate. Impedance of the cells reflects changes in cellular parameters such as cell proliferation, morphological changes (for example, spreading, adherence) and cell death, and is expressed as an arbitrary unit called the cell index (CI).

A total of 7,500 adherent IECs cells per well were added to a 96-well micro-E-plate to monitor the impedance of the cells every 3 min for 60 h. As the initial adherence in different wells can differ slightly, the CI was normalized to 1 after having reached the linear growth phase. After 24 h, freshly purified *S. cerevisiae*- and *M. restricta*-reactive CD154^+^CD69^+^ T_mem_ cells were added at different effector-to-target ratios (1:1, 1:2, 1:4). T cells and IECs were cross-linked by the addition of 1 μg ml^−1^ of SEB. T cell-induced lysis of IECs is indicated by the loss of impedance, that is, a decrease in the normalized CI. As a positive control, IECs were treated with 1% Triton X-100 (final concentration). For analysis with RTCA software (v.2.0.0.1301, ACEA), the mean of Triton X-100 samples was calculated and defined as 100% lysis after the addition of effector cells. The percentage of lysis for each sample was calculated in relation to a control sample without effector cells.

### Orthotopic TCR replacement in primary human CD4^+^ T cells

DNA templates (Supplementary Table 1) were designed in silico based on the previously published method^42^ and were synthesized by Twist Biosciences in pTwist Amp vectors. Briefly, the constructs comprise the full length of the α- and β-chains of the inserting TCRs flanked by left and right homology arms and contain self-cleaving peptides (P2A and T2A) to provide separation, as well as a poly(A) tail (bGHpA). The β-chain consists of the human variable region and the murine constant region, which is used as a tracking marker.

TCR replacement was performed as previously described^42^. In brief, CD4^+^ T cells were isolated by negative magnetic selection (CD4^+^ T Cell Isolation Kit; Miltenyi Biotec). Then, 4 × 10^7^ CD4^+^ cells were plated on 6-well cell-culture plates in expansion medium, containing TexMACS medium, supplemented with 5% (v/v) human AB-serum, 200 U ml^−1^ of IL-2 and 100 IU ml^−1^ of penicillin, 100 μg ml^−1^ of streptomycin and 0.25 μg ml^−1^ of amphotericin B. CD4^+^ T cells were subsequently activated for 2 d using the T Cell Activation/Expansion Kit (Miltenyi Biotec). Activated CD4^+^ T cells were harvested and the activation beads were removed using a MACSiMAG Separator (Miltenyi Biotec). For each nucleofection, 1 × 10^6^ cells were used and mixed with 20 μM ribonucleoprotein (RNP) mixtures (Integrated DNA Technologies (IDT)) and a homology-directed repair template (1 μg) (Twist Biosciences). The RNPs (all components from IDT) were generated in two sequential steps: first, the guide RNAs (gRNAs) were prepared by mixing equal volumes of *trans*-activating CRISPR (tracr)RNA (80 μM stock) with hTRAC CRISPR (cr)RNA (80 μM stock) (crRNA sequences for gRNAs were 5′-GGAGAATGACGAGTGGACCC-3′ for TRBC32, targeting both TRBC1 and TRBC2, and 5′-AGAGTCTCTCAGCTGGTACA-3′ for TRAC) and heating at 95 °C for 5 min, followed by cooling to room temperature (RT). In the second step, gRNAs were assembled with Cas9 nuclease by mixing equal volumes of Cas9 nuclease (6 μM) and gRNA (40 μM) and incubated at RT for 15 min. An electroporation enhancer was added (400 μM stock) to the RNP mixtures. The P3 Primary Cell 4D-Nucleofector X Kit S (Lonza) and a 4D-Nucleofector X unit (Lonza) with the EH100 program were used. Electroporated cells were immediately transferred to 96-well cell-culture plates in expansion medium and were incubated for 5 d at 37 °C and 5% CO_2_. After 5 d, TCR-transgenic CD4^+^ T cells were sorted with FACS on a FACS Aria Fusion (BD Bioscience) based on their mouse TCR-β expression and were further expanded for 2–3 weeks in the presence of 2 × 10^5^ irradiated allogeneic feeder cells until subsequent functional assays were performed. For FACS, cells were stained with fluorochrome-conjugated antibodies to CD4-Vioblue, CD8-PerCP, CD14-PerCP, CD20-PerCP (all Miltenyi Biotec) and mouse TCR-β chain-APC, as well as human TCR-α/β chain-FITC (both BioLegend). After sorting, knocked-in cells were expanded for 2 weeks.

### ScRNA-seq assay (10× Genomics)

For scRNA-seq and TCR-seq, yeast-reactive IL-17A producers, IFN-γ producers and double-negative CD154^+^ T_mem_ cells were sorted with FACS and labeled with barcoded antibodies (CITE-seq). In brief, PBMCs were stimulated with *C. albicans*, *C. tropicalis* and *S. cerevisiae* for 6 h in the presence of 1 μg ml^−1^ of CD40 and 1 μg ml^−1^ of CD28 pure antibody. Cells were labeled with IFN-γ and IL-17A catch reagent (cytokine secretion assay, both Miltenyi Biotec) and incubated for an additional 45 min in RMPI-1640 medium supplemented with 2% (v/v) human AB-serum under continuous rotation. Cells were further labeled with CD154-PE, IL-17A-APC and IFN-γ-FITC, followed by anti-PE MicroBeads, and magnetically enriched by MS Columns (all from Miltenyi Biotec).

Cells were sorted into precoated, low-bind collection tubes, containing sterile, filtered 1× PBS supplemented with 0.5% (v/v) human AB-serum. After sorting, cells were re-added to MS Columns to avoid cell loss during washing steps and stained on the column with l μl of hashtag antibodies (TotalSeq-C0251 anti-human hashtag 1 (LNH-94, 1:50), TotalSeq-C0252 anti-human hashtag 2 (LNH-94, 1:50) and TotalSeq-C0253 anti-human hashtag 3 (LNH-94, 1:50) in the presence of Human TruStain FcX in titer 1:20) (all BioLegend) in 50 μl of staining volume. Columns were washed twice and cells were eluted with 500 μl of sterile filtered 1× PBS supplemented with 0.5% (v/v) human AB-serum. Directly before loading, IL-17A^+^, IFN-γ and CD154^+^ populations for each antigen were combined, centrifuged, and loaded on a Chromium Next GEM Chip K (10× Genomics) according to the manufacturer’s instructions for processing with the Chromium Next GEM Single Cell 5′ Kit v.2. Between 5,000 and 12,000 cells were loaded for each reaction. TCR single-cell libraries were subsequently prepared from the same cells with the Chromium Single Cell V(D)J Enrichment Kit, Human T Cell (10× Genomics). Libraries were sequenced on an Illumina NovaSeq 6000 machine with 2× 100 bp for gene expression and TCR libraries, aiming for 50,000 reads per cell and 5,000 reads per cell, respectively.

### Single-cell transcriptome data analysis

The 10× sequencing data were processed using the 10× Genomics Cell Ranger v.6.0.0 software pipeline as previously described^56^. Cellranger mkfastq was used to demultiplex cellular barcodes and produce fastq files and cellranger count was used to map reads to the human genome (GRch38) with STAR aligner^57^ and obtain a matrix of transcript counts by cells.

The data analysis was performed with the scanpy Python package v.l.9.0, using Python v.3.7 (ref. 58). As part of the quality control, cells were subjected to the following criteria: (1) >500 expressed genes, (2) >1,000 total unique molecular identifier counts, (3) no doublets based on hashtag antibodies and (4) <5% of mitochondrial RNA. Out of the initial 63,732 cells, 60,130 satisfied these criteria and were used in the subsequent analysis.

The data normalization step included library size normalization to 10,000 counts per cell and log_2_(transformation) of the expression data. Highly variable genes were identified and used to reduce the dimensionality of the data to 30 principal components (PCs) with the principal component analysis algorithm. The PC values were adjusted using the harmony algorithm^59^ as implemented in scanpy.external. pp.harmony_integrate function to correct the batch effect introduced by differences in sample preparation. Unsupervised clustering of the cells was performed with the Leiden clustering algorithm (scanpy. tl.leiden) and cell subtypes were assigned to the clusters based on the differentially expressed marker genes.

### ScTCR repertoire data analysis

Cellranger vdj pipeline was used for V(D)J genes calling from scTCR-seq data. Scirpy Python package v.0.12.0 was used^60^ to integrate TCR data with the transcriptomic data, and TCR characteristics were inferred for each cell and defined the clonotypes. Cells were considered to belong to the same clonotype if they had the same TCR-β amino-acid sequence. As a quantitative measure of the clonality of TCR repertoires we calculated Gini’s coefficient^61^. Gini’s coefficient ranges from 0 to 1, with 0 corresponding to equal abundance of all clonotypes and values close to 1 representing total inequality (that is, high prevalence of one sequence). For Gini’s coefficient analysis, samples were normalized by random selection of 500 cells from each individual per fungal antigen to remove the impact of different sample sizes (number of cells per sample). TCR-sharing networks were created and visualized using the Cytoscape software v.3.9.1 (ref. 62). Clonotypes found in one cell only were excluded for network analyses.

### Trajectory inference

Pseudotime trajectory analysis was performed using the Monocle v.2 algorithm^35^. Samples were normalized by random selection of 500 cells from each individual per fungal antigen to remove the impact of different sample sizes (number of cells per sample) and the analysis restricted to the gene set of 36 markers (Extended Data Table 3).

### Statistical analysis

Statistical parameters including the exact value of *n*, the definition of center, dispersion and precision measure, and statistical significance are reported in the figures and figure legends. Statistical tests were performed with GraphPad Prism software v.9.4.1. Statistical tests were selected based on appropriate assumptions with respect to data distribution and variance characteristics; *P* < 0.05 was considered statistically significant.

### Software

Flow-cytometry data were analyzed using FlowJo v.10.9.0 (Tree Star) and Miltenyi MACSquantify v.2.13 (Miltenyi Biotec) software. Graphics and statistics were created with GraphPad Prism software v.9.4.1. TCR-sharing networks were created and visualized with Cytoscape software v.3.9.1 (ref. 62).

### Reporting summary

Further information on research design is available in the Nature Portfolio Reporting Summary linked to this article.

## Extended Data

**Extended Data Table 1:** Cohort characteristics

	Sample group			
	Healthy	IBD-FDR	UC	CD
Individuals	71	25	79	88
Sex, M/F (N, %)	31/40 (44/56%)	10/15 (40/60%)	41/38 (52/48%)	43/45 (49/51%)
Age (mean±SD, years)	37 ±13	55 ±18	42 ±15	43 ±14
ASCA IgG positive (N, %)	-	5 (20.0%)	1 (1.3%)	45 (51.1%)
ASCA IgA positive (N, %)	-	3(12.0%)	1 (1.3%)	46 (52.2%)
ASCA total positive (N, %)	0/0 (0.0%)	5 (20.0%)	2 (2.5%)	49 (55.6%)

**Extended Data Table 2:** Characteristics of patients with UC and CD

Montreal classification	Ulcerative colitis	Crohn’s disease
*Age at diagnosis in years*		
A1, <16, (N, %)	-	17 (19.3%)
A2, 17-40, (N, %)	-	51 (58.0%)
A3, >40, (N, %)	-	17 (19.3%)
Info not available, (N, %)	-	3 (3.4%)
*Location*		
L1, ileal, (N, %)	-	30 (34.1%)
L2, colonic, (N, %)	-	15 (17.1%)
L3, ileocolonic, (N, %)	-	39 (44.3%)
Info not available, (N, %)	-	4 (4.5%)
*Behaviour*		
B1, inflammatory, (N, %)	-	35 (39.8%)
B2, structuring, (N, %)	-	24 (27.3%)
B3, penetrating, (N, %)	-	25 (28.4%)
Info not available, (N, %)	-	4 (4.5%)
*Severity*		
S0, remission, no symptoms	17 (21.5%)	-
S1, mild symptoms	22 (27.9%)	-
S2, moderate symptoms	15 (19%)	-
S3, severe symptoms	5 (6.3%)	-
Info not available, (N, %)	20 (25.3%)	-
*Extensity*		
E1, ulcerative colitis	14 (17.7%)	-
E2, left-side UC; distal colitis	24 (30.4%)	-
E3, extensive colitis UC; pancolitis	21 (26.6%)	-
Info not available, (N, %)	20 (25.3%)	-
**Disease activity**		
*HBI*, mediandQR	-	2 ±5
Not active 0≤HBI≤4, (N, %)	-	57 (64.8%)
Mild activity, 5≤HBI≤7, (N, %)	-	16 (18.2%)
Moderate activity, 8≤HBI≤16, (N, %)	-	5 (5.7%)
Severe activity, 16≤HBI≤∞, (N, %)	-	1 (1.1%)
*pMayo score*, median±IQR	1d3	-
Remission <2 (N, %)	30 (38%)	-
Mild, 2-4 (N, %)	17 (21.5%)	-
Moderate, 5-7 (N, %)	5 (6.3%)	-
Severe, >7 (N, %)	0	-
Info not available, (N, %)	27 (34.1%)	9 (10.2%)
*CDAI*, median±IQR	-	60 ±84
*CAI*, median±IQR	2.5±4	-
*CRP*, mean±SD	7.7421.7	5.4 ±8.8
**Medication**		
Steroids, (N, %)	14 (17.7%)	11 (12.5%)
5-ASA, (N, %)	34 (43%)	11 (12.5%)
Infliximab, (N, %)	39 (49.3%)	52 (59.1%)
Adalimumab, (N, %)	3 (3.8%)	7 (7.9%)
Ustekinumab, (N, %)	3 (3.8%)	15 (17.1%)
Vedolizumab, (N, %)	18 (22.8%)	8 (9.1%)
Golimumab, (N, %)	1 (1.3%)	-
Tofacitinib, (N, %)	1 (1.3%)	-
No biological treatment, (N, %)	12(15.2%)	4 (4.5%)
Info not available, (N, %)	2 (2.5%)	2 (2.3%)

**Extended Data Table 3:** Gene set used for pseudotime trajectory analysis

Gene	UMAP population
*PRF1*	Th 1-cytotoxic
*GZMB*	Th 1-cytotoxic
*SLAMF7*	Th 1-cytotoxic
*PLEK*	Th 1-cytotoxic
*NKG7*	Th 1-cytotoxic
*CRTAM*	Th 1-cytotoxic
*CCL3*	Th 1-cytotoxic
*CCL4*	Th 1-cytotoxic
*CCL5*	Th 1-cytotoxic
*IFNG*	Th 1-cytotoxic
*TBX21*	Th 1-cytotoxic
*CSF2*	Effector/Th17
*IL2*	Effector/Th17
*IL21*	Effector/Th17
*IL22*	Effector/Th17
*IL17A*	Effector/Th17
*CCR7*	Tcm-Tem subsets
*SELL*	Tcm-Tem subsets
*CD27*	Tcm-Tem subsets
*ICOS*	Tfh/Tfr-like
*PDCD1*	Tfh/Tfr-like
*CXCR5*	Tfh/Tfr-like
*POU2AF1*	Tfh/Tfr-like
*CTLA4*	Tfh/Tfr-like
*LAG 3*	Tfh/Tfr-like
*IL10*	Tfh/Tfr-like
*MX1*	Type-I IFN signature
*MX2*	Type-I IFN signature
*ISG15*	Type-I IFN signature

## Supplementary Material

Supplementary Table 1

Reporting Summary

## Data Availability

Raw, paired-end, scRNA-seq fastq files from both gene expression and TCR libraries, as well as processed gene expression and TCR data as feature-barcode matrix files and clonotype tables, respectively, have been deposited at the Gene Expression Omnibus (accession no GSE227638) and GitHub under https://github.com/agbacher/fungi. All other data are available in pseudonymized form within 4 weeks upon request to the corresponding author.

## Code availability

All code is deposited in GitHub under https://github.com/agbacher/fungi.
