# Supplementary material for: Selection of cross-reactive T cells by commensal and food-derived yeasts drives cytotoxic TH1 cell responses in Crohn’s disease
Source: Nat Med. Author manuscript; Available in PMC 2023 Oct 18. (PMC10579100; doi:10.1038/s41591-023-02556-5)
Supplement: Reporting Summary [file EMS188949-supplement-Reporting_Summary.pdf]

nature portfolio

Corresponding author(s): Petra Bacher

Last updated by author(s): Aug 17, 2023

Reporting Summary

Nature Portfolio wishes to improve the reproducibility of the work that we publish. This form provides structure for consistency and transparency in reporting. For further information on Nature Portfolio policies, see our Editorial Policies and the Editorial Policy Checklist.

Statistics

For all statistical analyses, confirm that the following items are present in the figure legend, table legend, main text, or Methods section.

n/a	Confirmed	
☐	☒	The exact sample size (n) for each experimental group/condition, given as a discrete number and unit of measurement
☐	☒	A statement on whether measurements were taken from distinct samples or whether the same sample was measured repeatedly
☐	☒	The statistical test(s) used AND whether they are one- or two-sided<br>*Only common tests should be described solely by name; describe more complex techniques in the Methods section.*
☒	☐	A description of all covariates tested
☐	☒	A description of any assumptions or corrections, such as tests of normality and adjustment for multiple comparisons
☐	☒	A full description of the statistical parameters including central tendency (e.g. means) or other basic estimates (e.g. regression coefficient) AND variation (e.g. standard deviation) or associated estimates of uncertainty (e.g. confidence intervals)
☐	☒	For null hypothesis testing, the test statistic (e.g. F, t, r) with confidence intervals, effect sizes, degrees of freedom and P value noted<br>*Give P values as exact values whenever suitable.*
☒	☐	For Bayesian analysis, information on the choice of priors and Markov chain Monte Carlo settings
☒	☐	For hierarchical and complex designs, identification of the appropriate level for tests and full reporting of outcomes
☒	☐	Estimates of effect sizes (e.g. Cohen's d, Pearson's r), indicating how they were calculated

Our web collection on statistics for biologists contains articles on many of the points above.

Software and code

Policy information about availability of computer code

Data collection	Flow Cytometry data was acquired using LSR Fortessa (BD Biosciences) and Miltenyi MACSquantify software v2.13. For in vitro expansion cultures and for sequencing experiments, T cells were sorted using a FACS Aria II (BD Biosciences). Sequencing libraries were sequenced on a NextSeq6000 sequencer (Illumina). For measuring the killing capacities against epithelial cells data was acquired in xCELLigence RTCA Analyzer (Agilent).
Data analysis	Flow Cytometry data was analyzed using FlowJo v 10.9.0. Statistical analysis was performed using GraphPad Prism v9.4.1. Analysis of sequencing data was performed as detailed in the methods section, using the following software packages:<br>Cytoscape 3.9.1<br>Cell Ranger Version 6.0.0<br>Python v3.7<br>Scanpy 1.9.0<br>Scirpy 0.12.0<br>Monocle v2<br>RTCA software v 2.0.0.1301<br>Single cell analysis code can be found in GitHub under https://github.com/agbacher/fungi.

For manuscripts utilizing custom algorithms or software that are central to the research but not yet described in published literature, software must be made available to editors and reviewers. We strongly encourage code deposition in a community repository (e.g. GitHub). See the Nature Portfolio guidelines for submitting code & software for further information.

Data

Policy information about availability of data

All manuscripts must include a data availability statement. This statement should provide the following information, where applicable:

- Accession codes, unique identifiers, or web links for publicly available datasets
- A description of any restrictions on data availability
- For clinical datasets or third party data, please ensure that the statement adheres to our policy

Raw paired-end scRNASeq fastq files from both gene expression and TCR libraries, as well as processed gene expression and TCR data as feature-barcode matrix files and clonotype tables, respectively, have been deposited at GEO (accession number GSE227638). Custom code to reproduce the single RNA and TCR seq analysis under https://github.com/agbacher/fungi. All other data are available in pseudonymized form upon request to the corresponding author.

Human research participants

Policy information about studies involving human research participants and Sex and Gender in Research.

Reporting on sex and gender	Information on sex of the three cohorts was collected and reported in the Extended data Table 1.<br>Gender information was not collected for any of the cohorts.
Population characteristics	This study involved: 167 IBD patients, 25 First-degree-relatives and 71 healthy donors. Population characteristics are described in Extended data Table 1.<br>88 Crohn's Disease: age mean 43, Standard deviation +- 14, sex: M/F 43/45<br>79 Ulcerative Colitis: age mean 42, Standard deviation +- 15, sex: M/F 41/38<br>25 First-degree-relatives: age mean 55, Standard deviation +-18, sex: M/F 10/15<br>71 Healthy donors: age mean 37, Standard deviation +- 13, sex: M/F 31/40
Recruitment	Healthy donors were randomly recruited with the exclusion factors that they were not aware of having any gastrointestinal diseases or being first degree relatives of IBD patients. First-degree-relatives of IBD patients were recruited from the Kiel IBD Family Cohort Study, a German-wide cohort of IBD patients and their family members. Inflammatory bowel diseases patients were recruited at the Comprehensive Center for Inflammation Medicine (CCIM) and the Gastroenterology-Hepatology Center Kiel (GHZ-Kiel) based on their disease diagnosis and their willingness to give a sample. The CCIM and GHZ-Kiel doctors recruited patients on their schedule routine consultation, we foresee that based on disease/well-being status of the patient, patients under severe disease activity would not be attending their routine consultation. We do not foresee any other recruitment-biases.
Ethics oversight	-Peripheral EDTA blood samples of healthy donors were obtained from blood bank donors of the Institute for Transfusion Medicine, UKSH Kiel, Germany or from in-house volunteers (ethics committee CAU Kiel D578/18, D427/19).<br>-Peripheral EDTA blood samples from patients with inflammatory bowel diseases were obtained from the Department of Internal Medicine I, UKSH Kiel, the Comprehensive Center for Inflammation Medicine (CCIM) and the Gastroenterology-Hepatology Center Kiel (ethics committee CAU Kiel D427/19).<br>-Biopsies from inflamed and non-inflamed intestinal tissue were collected from CD patients that underwent colonoscopy (ethics committee CAU Kiel under the file: B 231/89-1/13).<br>- First-degree-relatives of IBD patients (IBD-FDRs) were recruited from the Kiel IBD Family Cohort Study, a German-wide cohort of IBD patients and their family members (ethics committee CAU Kiel A117/13), built by the popgen Biobank at Kiel University/University Hospital SH in Kiel.

Note that full information on the approval of the study protocol must also be provided in the manuscript.

Field-specific reporting

Please select the one below that is the best fit for your research. If you are not sure, read the appropriate sections before making your selection.

☒ Life sciences ☐ Behavioural & social sciences ☐ Ecological, evolutionary & environmental sciences

For a reference copy of the document with all sections, see nature.com/documents/nr-reporting-summary-flat.pdf

Life sciences study design

All studies must disclose on these points even when the disclosure is negative.

Sample size	The sample size was sufficient to demonstrate statistically significant differences in comparisons between experimental groups by the appropriate statistical tests. The sample size was also determined to be adequate based on reproducibility between independent experiments.
Data exclusions	No data were excluded from the analysis.

Replication

All of the experiments were successfully replicated, by measuring several individual donors of each cohort on different days in individual experiments. The data set includes a sufficient human sample size, taking into consideration the variability expected when using human samples.

Randomization

IBD patients were allocated into groups based on the disease diagnosis (Ulcerative colitis or Crohn's Disease). First-degree-relatives of IBD patients were allocated into the cohort based on their familial-association with IBD patients. Healthy donors were allocated into their group with the exclusion factors that they were not aware of having any gastrointestinal diseases or being first degree relatives of IBD patients. No additional randomization or group selection was performed.

Blinding

No blinding was performed due to patients being selected on their disease phenotype. First-degree-relatives experiments were not blinded due to selection based on their familial connection to IBD patients. Healthy donors experiments were not blinded due to in-house volunteer manner of collection.

Reporting for specific materials, systems and methods

We require information from authors about some types of materials, experimental systems and methods used in many studies. Here, indicate whether each material, system or method listed is relevant to your study. If you are not sure if a list item applies to your research, read the appropriate section before selecting a response.

Materials & experimental systems

n/a	Involved in the study
☐	☒ Antibodies
☒	☐ Eukaryotic cell lines
☒	☐ Palaeontology and archaeology
☒	☐ Animals and other organisms
☒	☐ Clinical data
☒	☐ Dual use research of concern

Methods

n/a	Involved in the study
☒	☐ ChIP-seq
☐	☒ Flow cytometry
☒	☐ MRI-based neuroimaging

Antibodies

Antibodies used

CD3-PE (clone: REA613) 1:50 - Cat#130-113-139
CD4-APC-Vio770 (clone: M-T466) 1:50 - Cat#130-113-251
CD4-VioBlue (clone: REA623) 1:50 - Cat#130-114-534
CD4-FITC (clone: REA623) 1:50 - Cat#130-114-585
CD8-VioGreen (clone: REA734) 1:50 - Cat#130-110-684
CD8-PerCP (clone: BW135/80) 1:50 - Cat#130-113-160
CD14-VioGreen (clone: REA599) 1:50 - Cat#130-110-525
CD14-PerCP (clone: TÜK4) 1:50 - Cat#130-113-150
CD20-VioGreen (clone: LT20) 1:50 - Cat#130-113-379
CD20-PerCP (clone: LT20) 1:50 - Cat#130-113-376
Viobility 405/520 Fixable Dye (1:100) - Cat#130-110-207
CD45RA-VioGreen (clone: REA562) 1:50 - Cat#130-113-369
CD45RO-APC (clone: REA611) 1:50 - Cat#130-113-369
CD69-PE (clone: REA824) 1:50 - Cat#130-112-613
CD154-FITC (clone: REA238) 1:50 - Cat#130-113-612
CD154-APC (clone: REA238) 1:50 - Cat#130-113-610
TNF-α-PE-Vio770 (clone: cA2) 1:50 - Cat#130-120-492
CD197 (CCR7)-PE-Bio770 (clone: REA108) 1:50 - Cat#130-118-350
IL-17A-PE-Vio770 (clone: REA1063) 1:50 - Cat#130-118-248
IL-21-VioR667 (clone: REA1039) 1:50 - Cat#130-117-423
IL-21-PE (clone: REA1039) 1:50 - Cat#130-117-421
Perforin-PE (clone: REA1061) 1:50 - Cat#130-118-117
GM-CSF-APC (clone: REA1215) 1:50 - Cat#130-123-420
GM-CSF-PE-Vio770 (clone: REA1215) 1:50 - Cat#130-123-422
Integrin b7-PE-Vio770 (REAfinity) 1:50 - Cat#130-106-442
Anti-HLA-DR (clone: AC122) 100ug/mL- Cat#130-108-056
CD154 MicroBeads kit - Cat#130-092-658
CD14 MicroBeads, human - Cat#130-050-201
IL-17 Secretion Assay – Detection Kit - Cat#130-094-536
IFN-γ Secretion Assay – Detection Kit - Cat#130-090-433
CD28 pure – functional grade, human (clone: 15E8) - Cat#130-093-375
CD40 pure – functional grade, human (clone: HB14) - Cat#130-094-133
CD3 pure – functional grade, human (clone: OKT3) - Cat#130-093-387
Anti-HLA-DR (clone: LT43) 100ug/mL- Cat#BE0306
CD4-BV421 (clone: OKT4) 1:20 - Cat#317434
CD45RA-PE-Cy5 (clone: HI100) 1:60 - Cat#304110
IFN-γ-BV785 (clone: 4S.B3) 1:20 - Cat#502542
IL-2-BV605 (clone: MQ1-17H12) 1:20 - Cat#500332

IL-2-BV711 (clone: MQ1-17H12) 1:20 - Cat#500346
IL-10-PE-Dazzle-594 (clone: JES3-9D7) 1:20 - Cat#501426
IL-17A-BV605 (clone: BL168) 1:20 - Cat#512326
Granzyme-B-PerCP-Cy5 (QA16A02) 1:20 - Cat#372212
CD319-PE-Dazzle-594 (clone: 162.1) 1:20 - Cat#331812
TNF-α-BV650 (clone: MAb11) 1:20 - Cat#502938
anti-mouse-TCRb-APC (clone: H57-597) 1:20 - Cat#109212
anti-human-TCRαb-FITC (clone: IP26) 1:20 - Cat#306706
Human TruStain FcX 1:20 - Cat#422302
IL-17A-BV650 (clone: N49-653) 1:20 - Cat#563746
Integrin b7-BV650 (clone: FIB504) 1:20 - Cat#564285
CCL4 (MIP-1b)-AF770-(clone: D21-1351) 1:20 - Cat#561278
Ki-67-AF700 (clone: B56) 1:20 - Cat#561277
Ki-67-R718 (clone: B56) 1:20 - Cat#566963
Ki-67-BV480 (clone: B56) 1:20 - Cat#566172
IL-22-PerCP-eFluor710 (clone: IL22JOP) 1:100 - Cat#46722282
TotalSeq-C0251 anti-human Hashtag 1 (clone: LNH-94) Biolegend Cat#394661 (1:50)
TotalSeq-C0252 anti-human Hashtag 2 (clone: LNH-94) Biolegend Cat#394663 (1:50)
TotalSeq-C0253 anti-human Hashtag 3 (clone: LNH-94) Biolegend Cat#394665 (1:50)

Validation

All antibodies purchased were validated by their manufacturers in-house.
Miltenyi Biotec:
By using the three pillars of antibody validation: 1. Antibody reproducibility and consistency (Pure antibody products and lot-to-lot consistency performance); 2. Antibody specificity (Epitope competition assay, KO validation using targeted genome editing and RNAi knockdown); 3. Antibody sensitivity (Functional testing of every product prior to release, performance comparison & compatibility with fixation).
https://www.miltenyibiotec.com/DE-en/products/macs-antibodies/antibody-validation.html

BioLegend:
Flow cytometry reagents
Specificity testing of 1-3 target cell types with either single- or multi-color analysis (including positive and negative cell types)
Once specificity is confirmed, each new lot must perform with similar intensity to the in-date reference lot. Brightness (MFI) is evaluated from both positive and negative populations.
Each lot product is validated by QC testing with a series of titration dilutions.
TotalSeq™ Antibodies
Bulk lots are tested by PCR and sequencing to confirm the oligonucleotide barcodes. They are also tested by flow cytometry to ensure the antibodies recognize the proper cell populations.
Bottled lots are tested by PCR and sequencing to confirm the oligonucleotide barcodes.
https://www.biolegend.com/en-us/quality/product-development

BD Biosciences:
https://www.bdbiosciences.com/en-de/products/reagents/flow-cytometry-reagents/research-reagents/quality-and-reproducibility
1. Antibody specificity: All flow cytometry reagents are titrated on the relevant positive or negative cells.
2. Quality Control: Quality control testing of new, manufactured lots are performed side-by-side with a previously accepted lot as a control, helping to serve as a reference for comparison and assuring that performance of the new lot is both reliable and consistent.
3. Lot-to-lot consistency: Testing with prior batches as reference helps you obtain consistent results with the new batch relative to the previous batches.

Flow Cytometry

Plots

Confirm that:

☒ The axis labels state the marker and fluorochrome used (e.g. CD4-FITC).

☒ The axis scales are clearly visible. Include numbers along axes only for bottom left plot of group (a 'group' is an analysis of identical markers).

☐ All plots are contour plots with outliers or pseudocolor plots.

☒ A numerical value for number of cells or percentage (with statistics) is provided.

Methodology

Sample preparation

Peripheral blood mononuclear cells (PBMCs) were freshly isolated from EDTA blood on the day of blood donation by density gradient centrifugation (Biocoll, Biochrom, Berlin, Germany). 1-2x10e7 PBMCs were plated in RPMI-1640 medium (GIBCO), supplemented with 5% (v/v) human AB-serum (Sigma Aldrich, Schnelldorf, Germany) in 12-well cell culture plates and stimulated for 7 h in presence of 1 µg/mL CD40 and 1 µg/mL CD28 pure antibody (both Miltenyi Biotec, Bergisch Gladbach, Germany). 1 µg/mL Brefeldin A (Sigma Aldrich) was added for the last 2 h.

Instrument

LSR Fortessa, FACS ARIA II (both BD Biosciences), MAQS-Quant16, MAQS-QuantX (both Miltenyi Biotec), Northern Lights (Cytek Biosciences), xCELLigence RTCA Analyzer (Agilent), 10x Chromium (10X Genomics).

Software

Miltenyi MACSquantify software (v2.13) and FlowJo (v10.9.0.)

Cell population abundance	All experiments include cells that were magnetically enriched following the ARTE protocol as described in the methods. Frequencies of antigen-specific T cells were determined based on the cell count of CD154+ CD4+T cells after enrichment, normalized to the total number of CD4+ T cells applied on the column. For each stimulation, CD154+ background cells enriched from the non-stimulated control were subtracted.
Gating strategy	All recorded events were gated according to FSC and SSC as lymphocytes; single cells were further selected using FSC-H vs. FSC-A. Subsequently living cells were identified as CD8-, CD14- CD20- and Viobility 405/520 Fixable Dye negative gated against CD4-APC-Vio770 (Clone MT466). The differentially stimulated cells were labeled with CD4-antibody-based fluorescent barcodes, allowing multiplexed analysis of T cell reactivity against different species as shown in Figure S1A. Each CD4+ population was then gated on CD154-FITC/IFN-g-BV785 and further gated into CD45RA-PE-Cy5 negative. All phenotypic and functional markers were analyzed withing CD154+CD45RA- cells.

☒ Tick this box to confirm that a figure exemplifying the gating strategy is provided in the Supplementary Information.
